# scHeteroNet: A Heterophily‐Aware Graph Neural Network for Accurate Cell Type Annotation and Novel Cell Detection

**DOI:** 10.1002/advs.202412095

**Published:** 2025-03-05

**Authors:** Jiacheng Liu, Xingyu Fan, Chunbin Gu, Yaodong Yang, Bian Wu, Guangyong Chen, Chang‐Yu Hsieh, Pheng‐Ann Heng

**Affiliations:** ^1^ Department of Computer Science and Engineering The Chinese University of Hong Kong Hong Kong 999077 China; ^2^ Zhejiang Lab Hangzhou 311100 China; ^3^ College of Pharmaceutical Sciences Zhejiang University Hangzhou 310058 China

**Keywords:** graph neural network, novel cell detection, single cell sequencing

## Abstract

Single‐cell RNA sequencing (scRNA‐seq) has unveiled extensive cellular heterogeneity, yet precise cell type annotation and the identification of novel cell populations remain significant challenges. scHeteroNet, a novel graph neural network framework specifically designed to leverage heterophily in scRNA‐seq data, is presented. Unlike traditional methods that assume homophily, scHeteroNet captures complex cell–cell interactions by integrating information from both immediate and extended cellular neighborhoods, resulting in highly accurate cell representations. Additionally, scHeteroNet incorporates an innovative novelty propagation mechanism that robustly detects previously uncharacterized cell types. Comprehensive evaluations across diverse scRNA‐seq datasets demonstrate that scHeteroNet consistently outperforms state‐of‐the‐art approaches in both cell type classification and novel cell detection. This heterophily‐aware approach enhances the ability to uncover cellular diversity, providing deeper insights into complex biological systems and advancing the field of single‐cell analysis.

## Graphical abstract

scHeteroNet) is a novel graph neural network model that explicitly addresses heterophily in single‐cell sequencing data enabling accurate cell type annotation and novel cell type discovery. By leveraging heterophily‐aware message passing and novelty propagation mechanisms, scHeteroNet achieves superior performance in both cell annotation and detection of previously uncharacterized cell populations across diverse biological contexts.

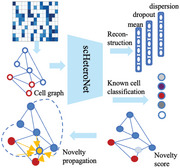

## 1 Introduction

Single‐cell RNA‐sequencing (scRNA‐seq) has revolutionized our ability to analyze the cellular composition of complex tissues, offering an unprecedented level of detail by measuring gene expression at the level of individual cell.^[^ ^1^, ^2^ ^]^, This technique has marked a significant advancement over traditional bulk RNA‐seq, enabling researchers to uncover the unique characteristics of each cell.^[^ ^3^ ^]^ Over the past decade, scRNA‐seq technologies have scaled to millions of cells, capturing cellular heterogeneity and variations, thus allowing researchers to delve into the intricate world within each cell.^[^ ^4^ ^]^ However, the analysis of scRNA‐seq data is not without challenges. The high dimensionality and the common occurrence of dropout events, where some gene expression data are not captured or are underrepresented, complicate data analysis.^[^ ^5^, ^6^ ^]^ Consequently, there is an urgent need for advanced computational methods capable of handling this high‐dimensional and sparse data.

A fundamental question in scRNA‐seq data analysis is how to computationally annotate cell types based on gene expression values. Existing methods for cell type annotation, such as Seurat,^[^ ^7^ ^]^ scVI,^[^ ^8^ ^]^ and scmap,^[^ ^9^ ^]^ primarily treat each cell as an isolated entity and employ methods designed for tabular data, thereby neglecting crucial relationships among cells (e.g., functional relationships).^[^ ^10^ ^]^ Graph‐based methods, such as Phenograph,^[^ ^11^ ^]^ utilize k‐nearest‐neighbor (KNN) graphs to model cell‐cell relationships. The emergence of graph neural networks (GNNs) has revolutionized the modeling of cell interactions.^[^ ^12^, ^13^, ^14^, ^15^, ^16^ ^]^ GNNs can extract features from the graph, providing detailed insights into cell‐cell relationships, enabling accurate separation of discrete cell clusters and reconstruction of cell trajectories.^[^ ^17^, ^18^, ^19^ ^]^ However, current research on GNN‐based cell annotation assumes homophily (connected cells are of the same type) in the constructed cell graph.^[^ ^20^, ^21^, ^22^, ^23^ ^]^ This assumption does not always hold true in many biological contexts, as different cell types can exhibit similar expression patterns due to shared biological functions or pathways, thereby limiting the accuracy of conventional methods. Furthermore, our experimental results challenge this assumption, revealing that it does not always hold and can lead to a substantial decline in performance (Results).

Moreover, current cell annotation approaches are generally inadequate for discovering novel cell types, as they tend to classify novel cells into preexisting categories within the reference dataset.^[^ ^24^, ^25^, ^26^, ^27^, ^28^, ^29^, ^30^, ^31^, ^32^ ^]^ This limitation hampers the identification of novel cells, which is crucial for uncovering meaningful biological insights.^[^ ^3^ ^]^ While out‐of‐distribution detection (OOD) techniques have been employed to discover new patterns,^[^ ^33^ ^]^ these techniques are primarily designed for conventional models and struggle to address the complexities of the intricate cell‐cell graph.

To address these limitations, we propose scHeteroNet, a GNN‐based framework that can accurately classify cell types and identify novel cells. scHeteroNet explicitly addresses heterophily in single‐cell data analysis and prioritizes novel cell detection through dedicated designs. It utilizes a heterophily‐aware GNN as an encoder to extract crucial structural information from the scRNA‐seq gene expression count matrix and cell graph. This module can propagate and aggregate information of each cell in the heterophily cell graph. It effectively encodes the features independently and aggregates information from distant neighbors to handle potential heterophily in the cell graph. Furthermore, scHeteroNet employs a heterophily‐aware novelty propagation method to derive the desired novelty score, which effectively identifies novel cells. Additionally, a decoder network based on the zero‐inflated negative binomial (ZINB) model is applied to capture the distribution of highly sparse and over‐dispersed scRNA‐seq data. Through rigorous evaluation with real‐world data, scHeteroNet has demonstrated superior performance in both accurately classifying known cell types and detecting previously uncharacterized cells. This capability not only enhances the accuracy of cell type classification but also significantly improves our ability to discover and study new cellular forms. We also explore its ability to integrate different single‐cell datasets, showcasing its versatility and potential for advancing our understanding of cellular diversity and function.

## 2 Results

### 2.1 The scHeteroNet Framework

**Figure 1:**
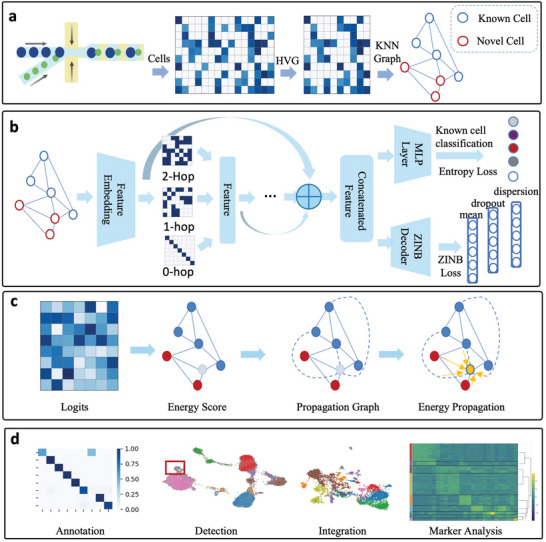
Overview of the proposed scHeteroNet. a) Construction of the cell graph. Sequencing results are preprocessed to select highly variable genes (HVGs), followed by graph construction using the KNN method. Some cells are known, while others are identified as novel. b) Heterophily‐aware cell annotation. scHeteroNet employs a heterophily‐aware graph neural network to learn from cell graphs. It utilizes information from multiple hops and the residual information. In addition to the conventional cross‐entropy loss, it also uses auxiliary loss conforming to a ZINB distribution. c) Heterophily‐aware novel cell detection. Based on the learned logits, scHeteroNet utilizes a novelty propagation process within the cell graph to identify novel cells. d) Downstream applications of scHeteroNet.

scHeteroNet is a graph‐based framework designed to annotate cell types and detect novel cells in scRNA data. It utilizes supervised learning to map gene expression data to cell types, transforming the complex, high‐dimensional expression data into a more manageable, lower‐dimensional feature space. As shown in **Figure** **1**, scHeteroNet features two primary components: one for known cell classification and the other for novel cell detection. Initially, scHeteroNet preprocesses gene expression data by filtering out low‐quality cells and genes, followed by selecting high‐variation genes (HVGs). Subsequently, it constructs a cell‐cell graph using the KNN method, where nodes represent individual cells and edges denote neighboring relationships based on proximity in gene expression space (Figure 1a).

Then, the scHeteroNet leverages a heterophily‐aware graph neural network to analyze the constructed cell graph. Unlike traditional GNNs that assume homophily (similarity between connected nodes), scHeteroNet is specifically designed to handle heterophily, where connected nodes can represent different cell types. This capability is particularly critical in biological contexts where neighboring cells can differ significantly in their functions. To better capture information from homogeneous neighbors, scHeteroNet incorporates neighbors from additional hops and separates the representation of own features from neighbors. Additionally, it uses the cross‐layer feature aggregation modular to aggregate features from different layers, enhancing feature integration across the network (Experimental Section). The model is trained using a combination of conventional cross‐entropy loss and an auxiliary loss that adheres to a ZINB distribution. This approach enhances scHeteroNet's ability to handle the over‐dispersed nature of biological data effectively (Figure 1b).

Despite scHeteroNet's ability to generate accurate labels, it is common in single‐cell analysis to encounter samples with unknown cell types, which are crucial for scientific discoveries. However, the performant novel cell detection ability often comes with compromised performance in cell classification. In scHeteroNet, we utilize the heterophily‐aware novelty propagation to help identify novel cells within the graph. Inspired by the works to establish an equivalence between neural classifier and energy‐based model, this approach can achieve highly accurate novel cell detection without influencing the annotation performance. Meanwhile, the novelty propagation method also follows a message‐passing process in the graph under the homogeneous assumptions. Thus, we propose constructing a propagation graph that includes distant neighbors, which may be homogeneous and thereby facilitate the novelty propagation process for detecting novel cells (Figure 1c). Finally, we can apply scHeteroNet for diverse downstream applications, including cell type annotation, novel cell detection, etc.(Figure 1d).

Overall, scHeteroNet's integration of these advanced techniques into a cohesive framework allows it to excel in the analysis of complex biological data, particularly in identifying and annotating heterogeneous and novel cell populations with high precision and efficiency.

### 2.2 scHeteroNet can Accurately Annotate Cell Types in Heterophily Cell Graph Across Multiple Datasets

We evaluated scHeteroNet against several state‐of‐the‐art methods, including ACTINN,^[^ ^34^ ^]^ CellTypist,^[^ ^35^ ^]^ scmap‐cell, scmap‐cluster,^[^ ^9^ ^]^ SCANVI,^[^ ^36^ ^]^ scBalance,^[^ ^37^ ^]^ GCN,^[^ ^38^ ^]^ scGraphFormer,^[^ ^39^ ^]^ scSimGCL,^[^ ^40^ ^]^ and scGCC^[^ ^41^ ^]^ using multiple scRNA‐seq datasets^[^ ^42^, ^43^, ^44^, ^45^, ^46^, ^47^, ^48^, ^49^, ^50^, ^51^, ^52^, ^53^ ^]^ to assess its performance in annotating cell types. These methods and datasets represent a range of advanced single‐cell annotation techniques and a diverse array of biological conditions and experimental settings.

**Figure 2:**
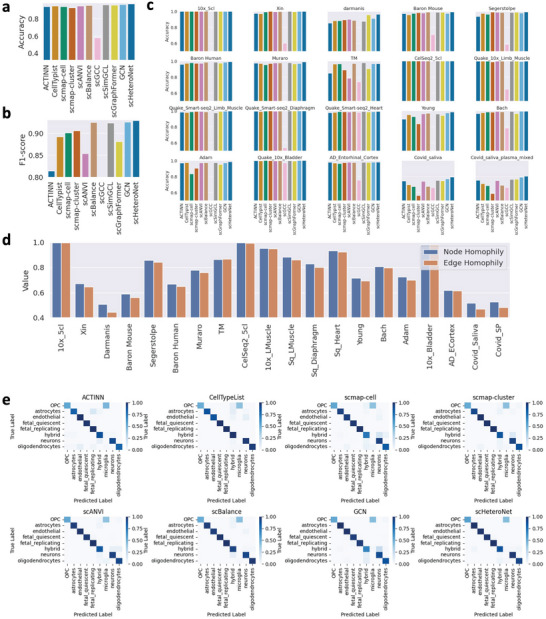
scHeteroNet can accurately annotate the known cells. a) Average accuracy of scHeteroNet compared to state‐of‐the‐art methods. b) Average F1 score of scHeteroNet compared to state‐of‐the‐art methods. c) Accuracy Comparisons between scHeteroNet and 10 single‐cell annotation methods on 20 scRNA‐seq datasets. d) Node homophily score and edge homophily score of the selected 20 datasets. e) Confusion matrix illustrating predictions of different methods on the Darmanis dataset. OPC stands for Oligodendrocyte Precursor Cells.

We assessed each method's performance using accuracy and F1‐score as metrics. The results revealed that scHeteroNet consistently surpassed the other methods, achieving the highest mean accuracy of 0.9711 (**Figure** **2**a) and mean F1‐scores up to 0.929 (Figure 2b) across the datasets. This indicates scHeteroNet's robustness and effectiveness in various biological contexts. The superior performance of scHeteroNet in accurately annotating cell types can be attributed to its heterophily‐aware message passing mechanism and cross‐layer feature aggregation. These innovative aspects of the algorithm design enable scHeteroNet to effectively handle complex cell graphs with heterophily, where connected cells may belong to different types. By capturing both local and extended neighborhood features, scHeteroNet learns accurate cell embeddings that reflect the true biological variability and complexity of the data. Detailed performance analysis across various datasets, such as 10x_5cl, Xin, Darmanis, Baron–Mouse, and Segerstolpe, further underscores scHeteroNet's superior performance (Figure 2c). scHeteroNet maintained high performance in these datasets. This is crucial for precise cell type annotation in complex biological systems characterized by heterophily, where different cell types exhibit less similarity in their expression profiles.

We also evaluated the node and edge homophily of each dataset (shown in Figure 2d). It is evident that datasets such as 10x_5cl, CeiSeq2_5cl, and 10x_Bladder exhibit high homophily scores for both nodes and edges, indicating that similar cell types are well‐connected in these datasets. Conversely, datasets like Darmanis, AD_ECortex, and Covid_SP show significantly lower homophily scores, suggesting a higher degree of heterophily where different cell types are intermixed. This variation in homophily levels across datasets demonstrates the diverse biological complexities and highlights the need for robust annotation methods like scHeteroNet that can effectively handle both homophilous and heterophilous structures. For instance, on the Covid_Saliva dataset, which is characterized by low homophily scores, the results indicate a heterophily graph. scHeteroNet achieves the best performance with a score of 0.794, which is 3.06% higher than the second‐best score of 0.771. In the Baron_Mouse and Segerstolpe datasets, scHeteroNet adeptly managed environments with both high and low homophily, displaying versatile performance that was not dependent on the level of homogeneity in the graph structures. This versatility confirms scHeteroNet's robustness and adaptability across a range of biological contexts. These results underscore scHeteroNet's capability to effectively capture and utilize heterophily in the data, a key factor for accurate cell type differentiation in heterophilous graph structures.

To provide a comprehensive understanding of the classification capabilities, Figure 2e displays confusion matrices for scHeteroNet and the other 7 representative algorithms, offering a detailed breakdown of prediction accuracy across various cell types such as OPCs, astrocytes, endothelial cells, fetal quiescent, fetal replicating, high neurons, and oligodendrocytes. scHeteroNet not only achieved the highest overall accuracy but also excelled in precision and recall for each specific cell type, demonstrating exceptional precision in distinguishing similar neuroectodermal derivatives like OPCs and astrocytes, high recall in identifying endothelial cells to minimize false negatives, and effective differentiation between quiescent and replicating fetal cells crucial for developmental studies. Additionally, scHeteroNet displayed high precision in distinguishing between functionally distinct cell types such as high neurons and oligodendrocytes. Compared to other methods such as scBalance, ACTINN, and CellTypist, scHeteroNet showed superior performance metrics, suggesting its algorithmic framework is better equipped to handle the subtleties of single‐cell RNA‐seq data. This accuracy enhances biological interpretations, crucial in complex tissues where precise cell type identification can lead to a better understanding of biological processes and disease mechanisms, highlighting scHeteroNet's reliability and technical robustness for researchers working with heterogeneous biological samples.

In summary, scHeteroNet exhibits outstanding performance in cell type annotation across a broad spectrum of datasets and biological conditions. Its ability to adeptly handle the complexities of cellular heterogeneity with a heterophily‐aware approach makes it a valuable tool for advancing our understanding of diverse biological systems.

### 2.3 scHeteroNet Enables Effectively Identify Novel Cells

**Figure 3:**
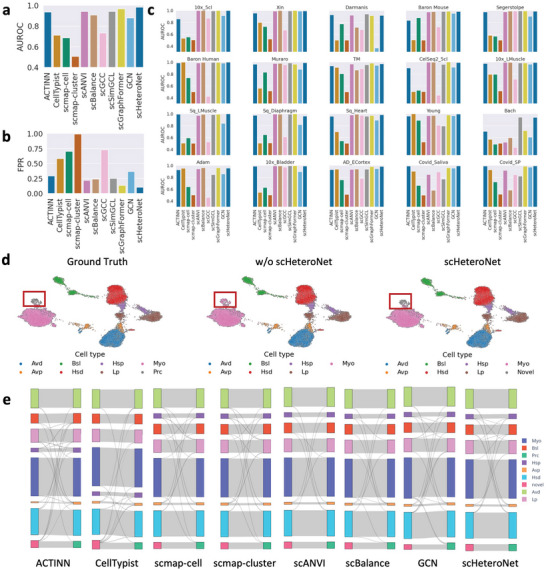
scHeteroNet can effectively identify the novel cells. a) Average AUROC of scHeteroNet compared to state‐of‐the‐art methods. b) Average FPR of scHeteroNet compared to state‐of‐the‐art methods. c) Comparisons of AUROC values between scHeteroNet and 10 single‐cell annotation methods across 20 real scRNA‐seq datasets. d) Annotation of Bach dataset, scHeteroNet can effectively identify novel cell types instead of incorrectly classifying them as known cell types. “w/o scHeteroNet” indicates that when the proposed model does not incorporate the novel cell detection functionality, it tends to incorrectly classify the unseen cell type as Myo. e) Sankey plot depicting the prediction outcomes in the Bach dataset. Avd stands for Differentiated Alveolar cells. Bsl stands for Basal cells. Hsp stands for Hormone sensing progenitors. Myo stands for Myoepithelial cells. Avp stands for Progenitors Alveolar cells. Hsd stands for Hormone sensing differentiated cells. Lp stands for Luminal progenitor cells. Prc stands for Protein C Receptor‐positive cells.

In our study, we rigorously assessed the efficacy of scHeteroNet in identifying novel cell types by benchmarking it against other leading computational techniques using a diverse set of established scRNA‐seq datasets. The summarized results, as depicted in **Figure** **3**, unequivocally demonstrate scHeteroNet's exceptional performance in novel cell type detection.

Figure 3a illustrates the performance metrics, specifically the Area Under the Receiver Operating Characteristic (AUROC). scHeteroNet consistently outperformed other methods across all datasets. On the 10x_5cl dataset, scHeteroNet achieved an AUROC of 0.999, surpassing GCN by 9.3% and ACTINN by 16.6%. The performance gap was also pronounced on the Bach dataset, where scHeteroNet attained an AUROC of 0.939, with the improvement being 34% and 55.8% compared to ACTINN and GCN, respectively. These significant improvements demonstrate scHeteroNet's superior precision and reliability in detecting novel cell types. For instance, in the Muraro dataset, scHeteroNet's AUROC is 0.989, which achieved the highest performance. These results consistently show that scHeteroNet substantially outperforms existing algorithms, with an average improvement of 28.2% across all datasets tested. Additionally, we found that while methods like CellTypist perform strongly in known cell annotation but poorly in novel cell detection, our method consistently demonstrates good performance in both annotation and novel cell detection.

Based on the false positive rate (FPR) results across various datasets, scHeteroNet demonstrates strong performance compared to other cell‐type annotation methods. In Figure 3b, the FPR metric is critical for understanding the rate at which the model incorrectly identifies non‐target cell types as novel ones. scHeteroNet achieves the lowest FPR on 6 out of 20 datasets, outperforming the second‐best method by an average of 36%. As for other datasets it's very close to the optimal metric value, e.g., on the 10x_5cl dataset, scHeteroNet's FPR is 0.001. For the Baron Mouse dataset, scHeteroNet attains an FPR of 0.071, surpassing ACTINN, CellTypist, and scmap‐cluster by 84.9%, 92.7%, and 92.9%, respectively. On average, scHeteroNet's FPR is 67.3% lower than the mean FPR of all other methods tested, demonstrating its robustness and reliability in cell type annotation tasks. Above all, these low FPR values highlight the model's effectiveness in reducing false discoveries, which is crucial for reliable novel cell type identification.

Further granularity is provided in Figure 3c, which compares AUROC values across multiple scRNA‐seq datasets including 10x_5cl, Xin, Darmanis, Baron_Mouse, and Segerstolpe. The AUROC scores were consistently above 0.9 across all datasets. This signifies a near‐perfect detection capability, essential for accurately identifying and characterizing new cell types in complex biological samples. The outstanding performance of scHeteroNet in identifying novel cells can be attributed to its innovative use of heterophily‐aware novelty propagation. By propagating novelty scores within the cell graph and incorporating a heterophilous adjustment term, scHeteroNet effectively distinguishes novel cells from known cell types. This novel approach leverages the structural information of heterophilous graphs, enabling accurate detection of novel cells even in challenging scenarios.

The Uniform Manifold Approximation and Projection (UMAP) visualizations in Figure 3d further illustrate the practical applications of scHeteroNet. The red boxes pinpoint areas where scHeteroNet has successfully identified novel cell types, distinctly separating these from known cell populations. This effective separation is crucial for downstream analyses and biological interpretation, ensuring that novel insights are based on accurate cell type identification.

Moreover, the Sankey diagrams in Figure 3e provide a visual representation of cell type distribution across the 8 selected methods. scHeteroNet's unique capability to identify novel cell types, which are depicted in bright pink, showcases its ability to detect cell populations that remain uncharacterized by other methods. This is particularly evident in its detection of rare cell types, which are often overlooked due to their low prevalence in samples.

In summary, scHeteroNet exhibits a notable capability to identify novel cell types across various scRNA‐seq datasets. Its superior performance metrics and effective visualization reinforce its potential as a powerful tool for discovering new cellular phenotypes, advancing our understanding of cellular heterogeneity and complexity in biological systems.

### 2.4 scHeteroNet Facilitates Effective Data Integration

Single‐cell atlases often include samples that span locations, laboratories and conditions, leading to complex, nested batch effects in data. In this section, we verified the ability of scHeteroNet to remove batch effects while retaining biological variation.

**Figure 4:**
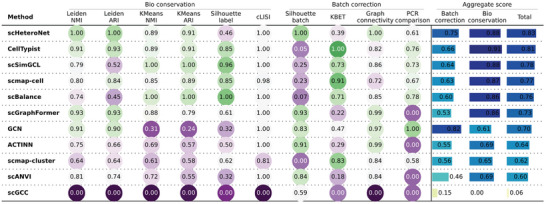
Benchmarking results for single‐cell integration based on the learned embedding using the Young dataset. This benchmark includes two types of tasks: biological conservation and batch correction.

**Figure** **4** presents a comprehensive comparison of several data integration metrics, including Leiden NMI (normalized mutual information), Leiden ARI (Adjusted Rand Index), KMeans NMI, KMeans ARI, Silhouette label, cLISI (cell‐type Local Inverse Simpson's Index), Silhouette batch, KBET (k‐nearest‐neighbor batch effect test), Graph connectivity, and PCR (Principal component regression) comparison. scHeteroNet achieved the highest scores in total metrics, indicated by its leading aggregate scores for batch correction and bio conservation. scHeteroNet's ability to effectively eliminate batch effects can be attributed to its graph structure and heterophily‐aware message‐passing design. By allowing flexible modeling of relationships that can differ from one batch to another, scHeteroNet captures essential relationships among cells, even in the presence of batch‐specific nuances. This innovative approach enables scHeteroNet to integrate datasets more effectively than baseline models, preserving biological integrity while minimizing batch effects.

**Figure 5:**
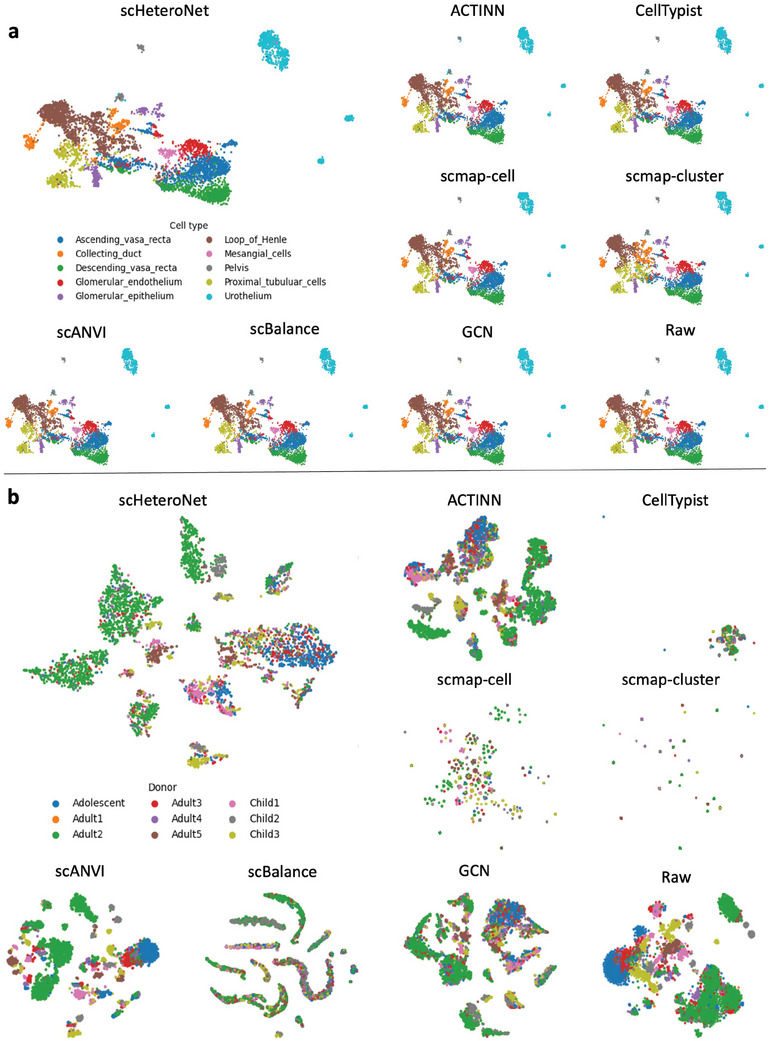
scHeteroNet can effectively integrate scRNA dataset. a) Visualization of the learned embedding for biological conservation in Young dataset. The t‐SNE plots display the embedding of cells colored by their cell types, showing well‐separated clusters for each cell type. This indicates that scHeteroNet can effectively capture and preserve the biological differences among cell types. b) Visualization of the learned embedding for batch correction in Young dataset. The t‐SNE plots show cells colored by donor batches, with the goal being to mix cells from different batches while maintaining biological integrity. The plots demonstrate a good mix of cells from different batches, indicating that scHeteroNet can effectively correct batch effects while preserving biological differences.

To better analyze the performance on different datasets. The t‐SNE (t‐distributed Stochastic Neighbor Embedding) visualizations in **Figure** **5**a,b display integrated datasets colored by cell type and donor, respectively. These visualizations compare scHeteroNet with other methods, highlighting scHeteroNet's superior performance in clustering different cell types and maintaining data integrity across batches. This is due to the heterophily‐aware message‐passing design that can effectively capture the essential relationships among different cells.

In Figure 5a, The t‐SNE plots show that scHeteroNet effectively integrates and clusters various cell types, such as Ascending_vasa_recta, Collecting_duct, Descending_vasa_recta, Glomerular_endothelium, Glomerular_epithelium, Loop_of_Henle, Mesangial_cells, Pelvis, Proximal_tubular_cells, and Urothelium, closely matching the ground truth. In contrast, other methods exhibit less distinct clustering and greater overlap between cell types. In Figure 5b, these t‐SNE plots illustrate the integration of data from different donors (Adolescent, Adult1, Adult2, Adult3, Adult4, Adult5, Child1, Child2, Child3). scHeteroNet demonstrates superior batch correction performance, maintaining the integrity of cell clusters across different donors and minimizing batch effects. Other methods show greater batch‐specific clustering, indicating less effective integration.

In summary, scHeteroNet excels in integrating scRNA‐seq datasets, effectively clustering cell types and correcting for batch effects. Its high performance across multiple integration metrics and superior visualization results make it a powerful tool for combining and analyzing single‐cell data from diverse sources, enhancing the reliability and interpretability of biological insights.

### 2.5 scHeteroNet can Help Identify Biologically Relevant Marker Genes

**Figure 6:**
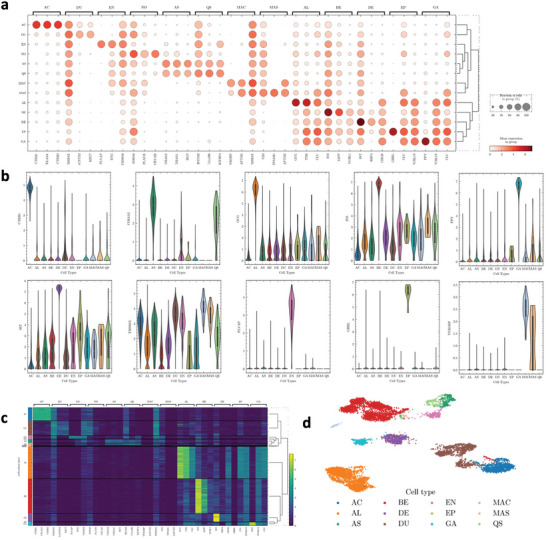
Marker gene analysis in the scRNA‐seq dataset Baron Human. a) Dot plot of average expression of the top five DEGs within each cell type. b) The violin plot of identified marker gene expression in the different cell types. c) The expression of each cell. d) The UMAP visualization of the dataset.

To validate the effectiveness of scHeteroNet's prediction results, we conduct a comprehensive evaluation of marker genes across different cell types. The results are displayed in **Figure** **6**, revealing the biological plausibility and relevance of the identified markers.

Figure 6a shows a heatmap and dot plot of marker gene expression across various cell types, including AC (Acinar cells), BE (Beta cells), DE (Delta cells), AS (Activated stellate cells), DU (Ductal cells), AL (Alpha cells), EP (Epsilon cells), GA (Gamma cells), EN (Endothelial cells), QS (Quiescent stellate cells), MAC (Macrophages), MAS (Mast cells), and NO (Novel cells). NO is the Schwann cell in the original dataset but it is not included in the training data. The size and color intensity of the dots represent the expression levels, highlighting key marker genes for each cell type. E.g., as we can see marker genes such as CTRB1, CTRB2, and CELA3A are strongly expressed in Acinar cells, consistent with their roles in digestive enzyme production and secretory function of the pancreas.^[^ ^54^ ^]^ In Endothelial cells, specific markers like PLVAP and ENG are highly expressed, indicating their involvement in vascular development and endothelial barrier function.^[^ ^55^ ^]^ Regarding Alpha cells, GCG is significantly expressed, reflecting its importance in glucagon production and glucose homeostasis.^[^ ^55^ ^]^ Intriguingly, the detected novel cell, CRYAB is highly expressed which matches the characteristic of Schwann cell.^[^ ^55^ ^]^

Figure 6b presents violin plots illustrating the distribution of expression levels for selected marker genes across different cell types. These plots further confirm the specificity and relevance of the identified markers. As we can find CTRB1 is high expression in Acinar cells, consistent with its role in digestive enzyme production.^[^ ^54^ ^]^ PPY elevated expression in Gamma cells, highlighting its function as the gene encoding pancreatic polypeptide, a key regulatory hormone that modulates pancreatic secretion and gastrointestinal function.^[^ ^56^ ^]^ GHRL predominant expression in Epsilon cells, aligning with its role in encoding ghrelin, a crucial orexigenic hormone that regulates energy homeostasis, appetite stimulation, and glucose metabolism.^[^ ^57^ ^]^

In summary, scHeteroNet excels in identifying biologically relevant marker genes across various cell types. These markers are consistent with known functions and characteristics of the respective cell types, demonstrating scHeteroNet's potential for uncovering critical biological insights in single‐cell RNA‐sequencing datasets.

### 2.6 scHeteroNet Enhance Novel Cell Detection Through Novelty Propagation

**Figure 7:**
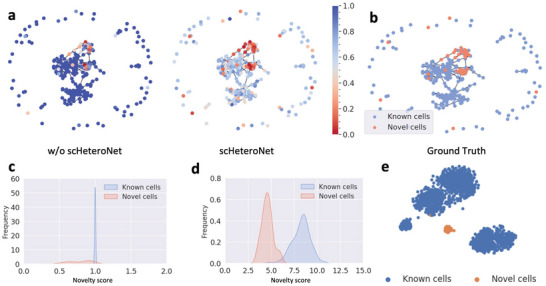
Novelty propagation enhances novel cell identification in a cell graph (Xin dataset). a) Visualization of the cell graph (for enhanced visual clarity, only the test set and the novel cell set are visualized) colored by novelty score, where a low score indicates a novel cell (red: low, blue: high). b) Cell graph highlighting known (blue) and novel (red) cells. c) Initial distribution of novelty scores for known and novel cells. d) Novelty score distribution after adjustment, showing improved separation between known and novel cells. e) UMAP projection of known (blue) and novel (orange) cells, corroborating the novelty score‐based classification.

Our study demonstrates the effectiveness of novelty propagation in identifying novel cell types within complex cell graphs. **Figure** **7** illustrates how this approach distinguishes known cell populations from potentially novel ones. Novelty propagation plays a crucial role in scHeteroNet's ability to identify novel cells. The novelty scores, which capture the likelihood of a cell being out‐of‐distribution, are propagated within the cell graph, taking into account both the cell's own novelty and the novelty of its neighbors. By considering the novelty scores of a broader neighborhood, scHeteroNet effectively distinguishes novel cells from known cell types. The propagation process allows the model to leverage the structural information of the graph, making it particularly well‐suited for detecting novel cells in heterophilous datasets.

Figure 7a presents a visualization of the cell graph, where each node represents an individual cell. The color gradient from red to blue indicates the novelty score assigned to each cell, with red signifying a lower score and a higher likelihood of being a novel cell type. This initial visualization provides an intuitive overview of how novel cells are distributed within the cell graph.

Building on this foundation, Figure 7b offers a clear binary classification of cells. Known cells are highlighted in blue, while novel cells are shown in red. This representation allows for a straightforward assessment of the spatial relationships between known and novel cell populations within the cell graph.

The distribution of novelty scores for both known and novel cells is depicted in Figure 7c. This initial distribution demonstrates the method's ability to assign different novelty scores to known and novel cells. However, some overlap between the two populations is evident, indicating room for improvement.

Figure 7d showcases the enhanced separation achieved through our novelty propagation technique. After adjustment, the novelty score distributions for known and novel cells show a more pronounced separation. This improved distinction underscores the power of our approach in refining the identification of novel cell types.

To further validate our findings, Figure 7e presents a UMAP visualization of the cells. This dimensional reduction technique corroborates the novelty score‐based classification by clearly separating known cells (blue) from novel cells (orange) in a different visualization space.

Collectively, these results highlight the robustness of our novelty propagation method in identifying novel cell populations within complex single‐cell datasets. By leveraging graph structure and novelty scores, our approach offers a powerful tool for discovering previously uncharacterized cell types, potentially leading to new insights in various biological contexts.

## 3 Discussion

In this study, we have introduced scHeteroNet, a computational framework developed to overcome significant challenges associated with the analysis of scRNA‐seq data. Traditional methods often struggle to effectively analyze the high‐dimensional and sparse nature of scRNA‐seq data and are typically inadequate in identifying novel cell types within these datasets. Our approach integrates a heterophily‐aware graph neural network with a zero‐inflated negative binomial distribution, specifically tailored to address the overdispersion commonly observed in single‐cell data, enhancing the robustness and accuracy of cell type annotation. While large models like scBert^[^ ^58^ ^]^ are good at general tasks and can utilize massive datasets to learn broad patterns, they often require substantial computational resources and may not capture the specific biological constraints and properties inherent in single‐cell data. In contrast, specialized architectures like scHeteroNet are valuable for targeted biological applications where domain‐specific inductive biases can improve performance.

A central innovation of scHeteroNet is its ability to handle heterophily effectively. In the constructed cell graph, it is common for connected nodes to exhibit dissimilar attributes; a scenario that poses significant challenges for conventional graph neural networks which typically assume homophily. The heterophily‐aware message passing mechanism of scHeteroNet is specifically designed to learn accurate cell embeddings that reflect the true biological variability and complexity, enabling the model to map intricate topological structures and relationships between cell types.

Furthermore, scHeteroNet excels in novel cell detection, a critical capability that many existing methods lack. It employs a novel heterophily‐aware novelty propagation process that ensures a robust detection of novel cell populations without sacrificing the accuracy of annotating known cell types. This dual capability was rigorously validated across a diverse array of scRNA‐seq datasets spanning multiple species, tissues, and technological platforms, where scHeteroNet consistently outperformed existing methods.

Looking ahead, the prospects for expanding scHeteroNet are both promising and extensive. The core principles of scHeteroNet are applicable not only to scRNA datasets, but also to other types of omics data like spatial transcriptomics. There is substantial scope for exploring alternative graph construction techniques that could potentially enhance the model's applicability and accuracy. Integrating additional biological knowledge could further refine the model's predictive capabilities, particularly in complex biological systems. Moreover, applying scHeteroNet to related analytical tasks such as trajectory inference could open new avenues for understanding cellular dynamics and developmental pathways. As single‐cell technologies advance and the volume and complexity of biological data continue to grow, the need for sophisticated analytical tools like scHeteroNet becomes increasingly critical. Such tools are essential not only for navigating the complexities of heterophily in biological datasets but also for driving forward the discovery of new biological insights.

In conclusion, scHeteroNet represents a significant advancement in the field of computational biology, offering a robust and flexible framework for the analysis of single‐cell data. By addressing the previously unmet challenge of heterophily in single‐cell analysis, this study not only enhances our understanding of cellular diversity but also sets a new benchmark for graph‐based models in computational biology, expanding the horizons for future research and applications.

## 4 Experimental Section

### Data Description

scHeteroNet's efficacy was rigorously assessed across a comprehensive array of scRNA‐seq datasets that were sequenced using diverse protocols and originated from various organs. Specifically, 20 datasets was compiled from publicly available sources (including 10x_5cl,^[^ ^42^ ^]^ Xin,^[^ ^43^ ^]^ Darmanis,^[^ ^44^ ^]^ Baron Mouse,^[^ ^45^ ^]^ Segerstolpe,^[^ ^46^ ^]^ Baron Human,^[^ ^45^ ^]^ Muraro,^[^ ^47^ ^]^ TM, CelSeq2_5cl,^[^ ^42^ ^]^ 10x_LMuscle, 10x_Bladde, Sq_LMuscle, Sq_Diaphragm, Sq_Heart,^[^ ^48^ ^]^ Young,^[^ ^49^ ^]^ Batch,^[^ ^50^ ^]^ Adam,^[^ ^51^ ^]^ AD ECortex,^[^ ^52^ ^]^ Covid Saliva, Covid SP^[^ ^53^ ^]^). scHeteroNet utilized the same datasets to validate its competence in identifying novel cells. Details of these datasets are provided in **Table** **1**. For each dataset 20% of data was used as test set, 20% data as the validation set. Then, the first class was randomly selected as unknown cell type. All the experiments are done with five random seeds (42, 66, 88, 2023, 2024).

**Table 1:** Overview of the original datasets for evaluation. These datasets encompass a diverse range of organs, employ various platforms.

Dataset	Organ	Cell	Gene	Class	Platform	Reference
10x_5cl	*Homo* lung	3803	11 778	5	10*x* *Genomics*	[42]
Xin	*Homo* pancreas	1449	33 889	4	SMARTer	[43]
Darmanis	*Mus* neuron	466	22 088	9	SMARTer	[44]
Baron Mouse	*Mus* pancreas	1886	14 861	13	inDrop	[45]
Segerstolpe	*Homo* pancreas	2133	22 757	13	Smart‐seq2	[46]
Baron Human	*Homo* pancreas	8569	17 499	14	inDrop	[45]
Muraro	*Homo* pancreas	2122	19 046	9	CEL‐seq2	[47]
TM	*Mus* *musculus*	54 865	19 791	55	10*x* *Genomics*	[^48^]
CelSeq2_5cl	*Homo* lung	570	12 627	5	CEL‐Seq2	[42]
10x_LMuscle	*Mus* Limb Muscle	3909	23 341	6	10*x* *Genomics*	[48]
10x_Bladder	*Mus* Bladder	2500	23 341	4	10*x* *Genomics*	[48]
Sq_LMuscle	*Mus* Limb Muscle	1090	23 341	6	Smart‐seq2	[48]
Sq_Diaphragm	*Mus* Diaphragm	870	23 341	5	Smart‐seq2	[48]
Sq_Heart	*Mus* Heart	4365	23 341	8	Smart‐seq2	[48]
Young	*Homo* Kidney	5685	33 658	11	10*x* *Genomics*	[49]
Bach	*Homo* Gammary Gland	23 184	19 965	8	10*x* *Genomics*	[50]
Adam	*Mus* Kidney	3660	23 797	8	Drop‐seq	[51]
AD_ECortex	*Homo* Entorhinal Cortex	42 528	19 965	8	10*x* *Genomics*	[52]
Covid_Saliva	*Homo* Saliva	14 502	27 714	13	10*x* *Genomics*	[53]
Covid_SP	*Homo* Plasma, Saliva	14 990	27 714	8	10*x* *Genomics*	[53]

### Data Preprocessing

The scRNA‐seq gene expression matrix is used as input for scHeteroNet model. Before inputting the expression profile into the model, a standard data processing step are conducted using the Scanpy package^[^ ^59^ ^]^ including data filtering, normalization, and log‐transformed. After that, the top 4000 highly variable genes were selected using the Seurat‐v3^[^ ^60^ ^]^ selection strategy due to the highly sparsity and rare non‐zero values in gene expression. Then the processed expression profiles are used as the feature of the input data into the model. The input feature is denoted as $X\in R^{v\times g}$, where *v* is the number of cells and *g* is the number of genes.

A cell graph was then constructed to present their relationships and the widely used KNN algorithm was adopted to construct the initial graph. In this graph, each node represents an individual cell, and the edges represent cellular similarities. Specifically, consider node *n*_*i*_ and node *n*_*j*_, there exists an edge between them if *n*_*i*_ is within the *k* nearest neighbor of *n*_*j*_. In the experimental setup, k was set to 15 and used Euclidean distance as the distance metric. The constructed cell graph is undirected with all edge weights set to 1.

Integrated with the graph, the final input data could be represented as G=(V,E), where V denotes the set of cell nodes and E denotes the set of edges connecting to each cell. For each cell node *n*_*i*_, its feature is represented as $X_{n_{i}}$.

### Evaluation Metrics

scHeteroNet was designed to simultaneously annotate cells and identify novel cell types. In this subsection, the evaluations were first overviewed in different contexts. Then introduce the main metrics in detail.

### 4.1 Evaluation Metrics—Evaluations in Different Contexts

In Section 2.2, accuracy was employed to evaluate the performance of each method on cell type annotation and introduced F1‐score as an additional metric to assess performance on imbalanced datasets, providing a more comprehensive evaluation of scHeteroNet's annotation capabilities. The homophily value of node and edge was used to evaluate the homophily of each dataset. The confusion matrix was also used to evaluate the annotation performance of scHeteroNet on each cell type. In Section 2.3, the area under the receiver operating characteristic curve (AUROC) and the false positive rate (FPR) were used to assess scHeteroNet's ability to identify novel cells. AUROC measures the overall performance of the model in distinguishing between novel and known cell types, with higher values indicating better discrimination. FPR, on the other hand, indicates the proportion of incorrect novel cell identifications among all negative samples, with lower values representing fewer false positives. In Section 2.4, batch correction and bio conservation are used for evaluating the performance of scHeteroNet on integrating scRNA‐seq dataset. And the overall score is calculated as (0.6*Bio) + (0.4*Batch).^[^ ^61^ ^]^ Detailed metric descriptions follow.

### Evaluation Metrics—Homophily Metrics

To assess the homogeneity of cell graphs, homophily metrics was employed,

**Node Homophily** Node homophily quantifies the extent to which nodes share the same label as their neighbors. It was defined as the average fraction of neighbors with the same label for each node, and can be expressed mathematically as:

(1) $$h_{node}=\frac{1}{\left|V\right|}\sum_{v_{i}\in V}\frac{\left|\{v_{j}|v_{j}\in N_{i},y_{j}=y_{i}\}\right|}{|N_{i}|}$$

here, V represents the set of all nodes, $N_{i}$ is the set of neighbors of node *v*_*i*_, and *y*_*i*_ is the label of node *v*_*i*_.

**Edge Homophily** Edge homophily measures the fraction of edges that connect nodes with the same label. It is defined as:

(2) $$h_{edge}=\frac{\left|\{\left(v_{i},v_{j}\right)\in E|y_{i}=y_{j}\}\right|}{\left|E\right|}$$

In this equation, E denotes the set of all edges in the graph.

### Evaluation Metrics—Cell Type Annotation Metrics

To evaluate the accuracy and robustness of cell type annotations, the following metrics were used:

**Accuracy**: The proportion of correctly annotated cells out of the total number of cells.

**F1 Score**: The harmonic mean of precision and recall, providing a balance between the two.

These metrics help in understanding how well scHeteroNet performs in annotating known cell types.

### Evaluation Metrics—Novel Cell Detection Metrics

For novel cell detection, which involves identifying OOD samples, several threshold‐independent metrics commonly used in the literature were employed:

**AUROC** This metric illustrates the trade‐off between the true positive rate (TPR) and false positive rate (FPR) across various thresholds. AUROC represents the probability that a positive example (in‐distribution sample) was assigned a higher score than a negative example (OOD sample). However, AUROC may not be ideal for imbalanced datasets.

**FPR** This metric indicates the probability of misclassifying an OOD sample as in‐distribution when the TPR is 95%. A lower FPR is desirable as it reflects fewer false positives at a high true positive rate.

### Compared with Existing Methods

To evaluate the performance of the proposed scHeteroNet against current scRNA‐seq annotation methods, a comprehensive comparison with several established techniques was conducted:

**ACTINN**: A method that automated identification of cell types in single‐cell RNA sequencing. ACTINN utilizes a neural network‐based approach to classify cell types based on scRNA‐seq data, providing an efficient and accurate annotation method.^[^ ^34^ ^]^

**CellTypist**: A method designed for cross‐species cell type classification. CellTypist leverages transfer learning techniques to annotate cell types by using pre‐trained models on well‐annotated datasets.^[^ ^35^ ^]^

**scANVI**: A method that extends the scVI framework by incorporating cell‐type labels in a semi‐supervised manner to improve cell‐type annotation accuracy.^[^ ^36^ ^]^

**scmap‐cluster and scmap‐cell**: These methods are part of the scmap framework, where scmap‐cluster assigns cells to the most similar cluster in a reference dataset, and scmap‐cell assigns individual cells based on the k‐nearest neighbors approach.^[^ ^9^ ^]^

**scBalance**: A sparse neural network‐based method targeting at imbalanced dataset. scBalance addresses the class imbalance problem in scRNA‐seq datasets by using adaptive weight sampling and dropout techniques to improve the annotation performance.^[^ ^37^ ^]^

**GCN**: This method employs graph convolutional networks for semi‐supervised classification. GCNs leverage the graph structure of the data, integrating information from neighboring cells to enhance the accuracy of cell‐type predictions.^[^ ^38^ ^]^

**scGraphFormer**: A transformer‐based GNN that learns a comprehensive cell‐cell relational network directly from scRNA‐seq data, demonstrating superior performance in cell type identification.^[^ ^39^ ^]^

**scSimGCL**: An innovative framework for scRNA‐seq data focused on cell clustering. It uses graph contrastive learning for self‐supervised pretraining, incorporates a unique graph structure learning mechanism, and has demonstrated superiority in clustering and imputation.^[^ ^40^ ^]^

**scGCC**: A novel graph self‐supervised contrastive learning model for scRNA‐seq data. It has a representation and clustering module and uses data augmentation and GAT.^[^ ^41^ ^]^

All of the methods are using their default parameters and settings. The data was only made pre‐process modifications to match the experiment datasets. Additionally, regarding the methods primarily proposed for clustering, namely scSimGCL and scGCC, the learned representation was utilize to train a classifier so as to make a fair comparison with the other methods. For scHeteroNet, a neural network architecture consisting of an initial feature extraction layer was implemented using a MLP layer with 32 neurons, followed by graph convolution layer with a hidden dimension of 32. Regarding the training parameters, the model was trained for 200 epochs, the optimization was performed using Adam optimizer with a learning rate of 1e‐2 and weight decay of 5e‐3. Additionally, the ZINB weight was set to 1e‐4 and the propagation parameter (β) to 0.5. All of the experiments were running on the workstation with Intel(R) Xeon(R) W‐2223 CPU @ 3.60GHz, Ubuntu 22.04, NVIDIA 4090 GPU and 64GB RAM.

### Heterophily‐Aware Graph Representation Learning in Cell Graph

A novel graph neural network model was proposed to classify the known cell types and detect novel cell types simultaneously. Consider a cell graph G=(V,E), where V denotes the set of nodes and E denotes the set of edges. The graph structure was represented by a binary adjacency matrix $A\in {\left\{0,1\right\}}^{\left|V|\times |V\right|}$. Each node *n*_*i*_ in the graph is associated with a feature vector $x_{n_{i}}$ and a label $y_{n_{i}}$ and the overall feature matrix and class vector can be represented by *X* and *y*, respectively. In the setting, the node set *V* can further split into two subsets, i.e., the known cells $E_{k}$ and the unknown cell set $E_{u}$. This work aims to classify the cells in $E_{k}$ to known cell types and detect cells that belong to $E_{u}$. Accordingly, the feature matrix and label vector can be divided into *X* = [*X*_*k*_, *X*_*u*_] and *y* = [*y*_*k*_, *y*_*u*_]. In this subsection, the focus was mainly on the cell annotation part, and the novel cell detection will be introduced in the next subsection.

### Heterophily‐Aware Graph Representation Learning in Cell Graph—Heterophily‐Aware Graph Neural Network

To effectively aggregate features in the cell graph, the heterophily‐aware graph neural network in scHeteroNet was introduce. This model was specifically designed to handle the challenges posed by heterophilous graphs, where nodes with different labels were often connected. Traditional graph neural networks tend to struggle in such scenarios due to their reliance on homophily assumptions. The approach leverages advanced message passing and aggregation techniques to overcome these limitations.^[^ ^62^, ^63^ ^]^

**Two‐hop message passing** Message passing was a fundamental operation in graph neural networks, where each node aggregates information from its neighbors to update its feature representation. In the context of heterophilous graphs, this process needs to be adapted to capture more extensive relational information, which may be present not only in immediate neighbors but also in nodes that are more hops away. In scHeteroNet, the traditional message‐passing framework was extended to include two‐hop neighbors. This extended message‐passing mechanism is crucial in heterophilous graphs because it allows the model to capture relevant information from nodes that are not directly connected but are two hops away. The two‐hop feature aggregation can be formulated as:

(3) $$h_{i}^{\left(l+1\right)}=\sigma \left(W_{0}h_{i}^{\left(l\right)}+\sum_{v_{j}\in N_{i}}W_{1}h_{j}^{\left(l\right)}+\sum_{v_{k}\in N\left(N_{i}\right)}W_{2}h_{k}^{\left(l\right)}\right)$$

where $h_{i}^{\left(l\right)}$ represents the feature vector of node *v*_*i*_ at layer *l*, $N_{i}$ denotes the set of direct neighbors of *v*_*i*_, $N(N_{i})$ denotes the set of two‐hop neighbors, and **W**_0_, **W**_1_, and **W**_2_ are learnable weight matrices. The term σ denotes an activation function. This formulation separates the self‐feature, direct neighbors' features, and two‐hop neighbors' features, allowing the model to differentially weight information from these distinct sources. The advantage of this separation is that it enables the model to better capture the complex dependencies in heterophilous graphs, improving the representation of node features.

**Cross‐layer feature aggregation** To capture long‐range dependencies and diverse neighborhood information, a cross‐layer feature aggregation mechanism was used. This technique allowed the model to adaptively select and combine features from different layers of the network, ensuring that both local and global information was incorporated into the final node representation. Mathematically, the feature representation of a node *v*_*i*_ at layer *l* can be expressed as:

(4) $$h_{i}^{\left(l\right)}=\gamma \left(\left\{h_{i}^{\left(k\right)}|k=0,\text{…},l\right\}\right)$$

where $h_{i}^{\left(k\right)}$ represents the feature vector of node *v*_*i*_ at layer *k*. γ is the aggregation function, in this work, concatenation was used as the aggregation operator. The cross‐layer aggregation mechanism ensures that the final node representation incorporates both local and global information, enhancing the model's ability to learn in heterophilous settings.

By integrating two‐hop message passing and cross‐layer feature aggregation, scHeteroNet effectively captures both local and extended neighborhood features, addressing the challenges of heterophilous graphs. The combined node features were then used for downstream tasks such as cell type annotation and novel cell detection.

### Heterophily‐Aware Graph Representation Learning in Cell Graph—ZINB‐Based Decoder

To effectively capture the inherent structure of scRNA‐seq data from the latent embedded representation *Z*, the ZINB model^[^ ^64^ ^]^ was integrated into the proposed scHeteroNet. This integration allows for a more accurate reconstruction of the data by accounting for both the overdispersion and the excess zeros commonly observed in scRNA‐seq datasets. The choice of the ZINB model was motivated by previous research, which has demonstrated that the distribution of scRNA‐seq data can be well‐approximated by a ZINB distribution.^[^ ^64^ ^]^ This probabilistic model captures the global structure of the data more effectively than simpler models, facilitating improved downstream analysis. The ZINB distribution is defined as follows:

(5) $$\begin{array}{cc} & \mathrm{ZINB}(X|\pi ,\mu ,\theta )\\ & =\pi \delta \left(X\right)+\left(1-\pi \right)\left[\frac{\Gamma (X+\theta )}{X!\Gamma (\theta )}{\left(\frac{\theta }{\theta +\mu }\right)}^{\theta }{\left(\frac{\mu }{\theta +\mu }\right)}^{X}\right] \end{array}$$

where μ and θ represent the mean and dispersion, respectively, and π is the weight of the point mass at zero. This formulation allows the model to handle both the zero‐inflation and the variability characteristic of scRNA‐seq data. To estimate the parameters {π, μ, θ} from the latent embedded representation *Z*, multi‐layer perceptron layers were append after the hidden state. These layers transform *Z* into the parameters of the ZINB distribution as follows:

(6) $$\begin{array}{c} \Pi =\mathrm{sigmoid}\left(W_{\pi }f_{D}\left(Z\right)\right)\\ M=\exp\left(W_{\mu }f_{D}\left(Z\right)\right)\\ \Theta =\exp\left(W_{\theta }f_{D}\left(Z\right)\right) \end{array}$$

where *f*_*D*_ denotes the MLP model, and *W*_π_, *W*_μ_, and *W*_θ_ are the learned weight matrices. The parameter matrices Π, *M*, and Θ correspond to the dropout probability, mean, and dispersion of the ZINB distribution, respectively. This parameterization ensures that the model captures the essential characteristics of the scRNA‐seq data.

### Training scHeteroNet on Cell Graphs

To train scHeteroNet effectively on cell graphs, a combination of cross‐entropy loss was employed for classification and the negative log‐likelihood of the ZINB distribution for reconstruction. These loss functions are weighted and summed to optimize the model.

For known cells, the commonly employed cross‐entropy loss was used as the classification loss function. Specifically, a classification head was added after the extracted features *Z* and calculate the loss with respect to the known cell types. The cross‐entropy loss is formulated as:

(7) $$L_{cls}=-\frac{1}{|V_{l}|}\sum_{i=1}^{|V_{l}|}\log\left(z_{i,y_{i}}\right)$$

where $V_{l}$ denotes the set of labeled nodes, and $z_{i,y_{i}}$ represents the predicted probability for the true class *y*_*i*_ of node *i*.

In addition to the classification loss, the negative log‐likelihood of the ZINB distribution was used as the reconstruction loss function for the original scRNA‐seq data *X*. This loss function is defined as:

(8) $$L_{\text{ZINB}}=-\log\left(\mathrm{ZINB}\left(X|\pi ,\mu ,\theta \right)\right)$$

where π, μ, and θ are the parameters of the ZINB distribution, learned from the latent representation *Z*. By minimizing this loss, the autoencoder learns to accurately reconstruct the scRNA‐seq data from the latent space, capturing the underlying data distribution and addressing the zero‐inflation and overdispersion challenges inherent in scRNA‐seq datasets.

Finally, the total loss is a weighted sum of these two components:

(9) $$L_{total}=L_{cls}+\alpha L_{\text{ZINB}}$$

where α is hyperparameters that balance the contributions of the classification and reconstruction losses. These weights are chosen to ensure that both tasks are effectively optimized during training.

In summary, the training of scHeteroNet on cell graphs involves optimizing a weighted combination of classification and reconstruction losses, enabling the model to learn to both classify cell types accurately and reconstruct the scRNA‐seq data.

### Heterophily‐Aware Novel Cell Detection

Once the scHeteroNet model is trained, it can effectively extract meaningful features from the sequencing results. Here, an energy‐based method was adopted to detect novel cells, which is effective and can be done independently of the annotation process.^[^ ^65^ ^]^ This is particularly important because joint training of an annotator and a detector can be complex and may negatively impact the performance of both models.

Existing methods for out‐of‐distribution detection in graph neural networks were built on the assumption of homophily, which may not hold true for most single‐cell datasets characterized by heterophily.^[^ ^66^, ^67^ ^]^ To address this, an OOD detection method tailored for heterophilous graphs was implemented.

First, the potential was quantified for novel cells by calculating a novelty score, which reflects the likelihood that a cell is novel. The novelty score for a cell *x* is calculated using the following equation:

(10) $$E\left(x,G_{x};h_{\theta }\right)=-\log\sum_{c=1}^{C}e^{h_{\theta }\left(x,G_{x}\right)\left[c\right]}$$

where *h*_θ_(*x*, *G*_*x*_) represents the logits output by the scHeteroNet model for cell *x* and its ego‐graph *G*_*x*_. The logits were obtained from the trained scHeteroNet model, which has been trained with a standard supervised classification loss on in‐distribution data.

Then, to effectively detect novel cells in heterophilous graphs, the novelty propagation was ran within two‐hop neighbors. This approach ensures that the novelty score incorporates information from a broader neighborhood, capturing the complex dependencies in heterophilous graphs. The novelty score for a cell *x* is calculated using the following function:

(11) $$E^{k}=\beta E^{k-1}+\left(1-\beta \right)\left(BE^{\left(k-1\right)}\right)$$

where β is a parameter controlling the balance between the novelty of the cell itself and the novelty propagated from its neighbors. *B* is the normalized two‐hop adjacency matrix of the graph.

In summary, our heterophily‐aware novelty‐based detection method leverages graph structural information to identify novel cells. By propagating novelty scores within two‐hop neighbors and incorporating a heterophilous adjustment term, our approach is well‐suited for single‐cell datasets with complex dependencies.

### Data and Code Availability

All datasets utilized in this study are publicly accessible as detailed in their original publications (see Table 1). No new data were generated specifically for this study. Meanwhile, all processed data was made available on Zenodo (https://zenodo.org/records/14633897). Additionally, an open‐source implementation of scHeteroNet is available on GitHub (https://github.com/mrbeann/scHeteroNet).

## Conflict of Interest

The authors declare no conflict of interest.

## Supporting information


Supporting Information

## Data Availability

The data that support the findings of this study are available from the corresponding author upon reasonable request.

## References

[1] J. Cao , J. S. Packer , V. Ramani , D. A. Cusanovich , C. Huynh , R. Daza , X. Qiu , C. Lee , S. N. Furlan , F. J. Steemers , A. Adey , R. H. Waterston , C. Trapnell , J. Shendure , Science 2017, 357, 661. 28818938 10.1126/science.aam8940 PMC5894354

[2] Method of the Year 2013 , Nat. Methods 2013, 11, 10.1038/nmeth.2801. 24524124

[3] V. Svensson , R. Vento‐Tormo , S. A. Teichmann , Nat. Protoc. 2018, 13, 599. 29494575 10.1038/nprot.2017.149

[4] C. Cheng , W. Chen , H. Jin , X. Chen , Cells 2023, 12, 1970. 37566049 10.3390/cells12151970 PMC10417635

[5] D. Molho , J. Ding , Z. Li , H. Wen , W. Tang , Y. Wang , J. Venegas , W. Jin , R. Liu , R. Su , P. Danaher , R. Yang , Y. L. Lei , Y. Xie , J. Tang , ArXiv 2022, *abs/2210.12385*.

[6] Y. Shan , J. Yang , X. Li , X. Zhong , Y. Chang , Inf. Sci. 2022, 621, 88.

[7] A. Butler , P. Hoffman , P. Smibert , E. Papalexi , R. Satija , Nat. Biotechnol. 2018, 36, 411. 29608179 10.1038/nbt.4096 PMC6700744

[8] R. Lopez , J. Regier , M. B. Cole , M. I. Jordan , N. Yosef , Nat. Methods 2018, 15, 1053. 30504886 10.1038/s41592-018-0229-2 PMC6289068

[9] V. Y. Kiselev , A. Yiu , M. Hemberg , Nat. Methods 2018, 15, 359. 29608555 10.1038/nmeth.4644

[10] Y. Gan , X. Huang , G. Zou , S. Zhou , J. Guan , Brief. Bioinform. 2022. 10.1093/bib/bbac018 35172334

[11] J. H. Levine , E. F. Simonds , S. C. Bendall , K. L. Davis , E. ad David Amir , M. D. Tadmor , O. Litvin , H. G. Fienberg , A. Jager , E. R. Zunder , R. Finck , A. L. Gedman , I. Radtke , J. R. Downing , D. Pe'er , G. P. Nolan , Cell 2015, 162, 184. 26095251 10.1016/j.cell.2015.05.047 PMC4508757

[12] Z. Zhang , P. Cui , W. Zhu , IEEE Trans. Knowl. Data Eng. 2022, 34, 249. 10.1109/tkde.2020.3045924 PMC10619966 37915376

[13] Z. Wu , S. Pan , F. Chen , G. Long , C. Zhang , P. S. Yu , IEEE Trans. Neural Netw. Learn. Syst. 2019, 32, 4. 10.1109/TNNLS.2020.2978386 32217482

[14] J. Zhou , G. Cui , Z. Zhang , C. Yang , Z. Liu , M. Sun , AI Open 2020.

[15] Z. Yu , Y. Su , Y. Lu , Y. Yang , F. Wang , S. Zhang , Y. Chang , K. chun Wong , X. Li , Nat. Commun. 2023, 14, 400. 36697410 10.1038/s41467-023-36134-7 PMC9877026

[16] Z. Yu , Y. Lu , Y. Wang , F. Tang , K. chun Wong , X. Li , Proc. Conf. AAAI Artif. Intell. 2022, 36, 4671.

[17] Y. Zeng , X. Zhou , Z. Pan , Y. Lu , Y. Yang , Brief. Bioinform. 2022, 23, bbab570.35018408

[18] X. Shao , H. Yang , Z. Xiang , J. Liao , P. Yang , J. Cheng , X. Lu , H. Chen , X. Fan , Nucleic Acids Res. 2021, 49, e122. 34500471 10.1093/nar/gkab775 PMC8643674

[19] J. Wang , A. Ma , Y. Chang , J. Gong , Y. Jiang , R. Qi , C. Wang , H. Fu , Q. Ma , D. Xu , Nat. Commun. 2020, 12, 1882. 10.1038/s41467-021-22197-x PMC7994447 33767197

[20] J. Zhu , Y. Yan , M. Heimann , L. Zhao , L. Akoglu , D. Koutra , IEEE Data Eng. Bull. 2023, 46, 12.

[21] J. Zhu , R. A. Rossi , A. B. Rao , T. Mai , N. Lipka , N. Ahmed , D. Koutra , Proc. Conf. AAAI Artif. Intell. 2020, 25, 11168.

[22] Z. Zhong , S. Ivanov , J. Pang , Trans. Mach. Learn. Res. 2022, 2022.

[23] Q. Huang , H. He , A. Singh , S.‐N. Lim , A. R. Benson , ICLR 2021.

[24] D. P. Lewinsohn , K. A. Vigh‐Conrad , D. F. Conrad , C. B. Scott , Bioinformatics 2023, 39, btad360. 37267208 10.1093/bioinformatics/btad360 PMC10272704

[25] V. Nguyen , J. Griss , BMC Bioinformatics 2022, 23. 10.1186/s12859-022-04574-5 PMC8762856 35038984

[26] J. C. Kimmel , D. R. Kelley , Genome Res. 2021, 31, 1781. 33627475 10.1101/gr.268581.120 PMC8494222

[27] Y. Tan , P. Cahan , Cell Syst. 2019, 9, 207. 31377170 10.1016/j.cels.2019.06.004 PMC6715530

[28] Y.‐X. Xiong , M.‐G. Wang , L. Chen , X.‐F. Zhang , PLoS Comput. Biol. 2023, 19, e1011261. 37379341 10.1371/journal.pcbi.1011261 PMC10335708

[29] J. Chen , H. Xu , W. Tao , Z. Chen , Y. Zhao , J.‐D. J. Han , Nat. Commun. 2023, 14. 10.1038/s41467-023-35923-4 PMC9840170 36641532

[30] S. Wang , A. O. Pisco , A. McGeever , M. Brbić , M. Zitnik , S. Darmanis , J. Leskovec , J. Karkanias , R. B. Altman , Nat. Commun. 2021, 12. 10.1038/s41467-021-25725-x PMC8455606 34548483

[31] D. Li , J. Ding , Z. Bar‐Joseph , Genome Res. 2022, 32, 1765. 35764397 10.1101/gr.276609.122 PMC9528981

[32] Q. Yin , Y. Wang , J. Guan , G. Ji , Brief. Bioinform. 2021, 23, bbab508. 10.1093/bib/bbab508 34913057

[33] H. Li , X. Wang , Z. Zhang , W. Zhu , arXiv:2202.07987 2022.

[34] F. Ma , M. Pellegrini , Bioinformatics 2020, 36, 533. 31359028 10.1093/bioinformatics/btz592

[35] C. Domínguez Conde , C. Xu , L. Jarvis , D. Rainbow , S. Wells , T. Gomes , S. Howlett , O. Suchanek , K. Polanski , H. King , L. Mamanova , N. Huang , P. A. Szabo , L. Richardson , L. Bolt , E. S. Fasouli , K. T. Mahbubani , M. Prete , L. Tuck , N. Richoz , Z. K. Tuong , L. Campos , H. S. Mousa , E. J. Needham , S. Pritchard , T. Li , R. Elmentaite , J. Park , E. Rahmani , D. Chen , et al., Science 2022, 376, eabl5197. 35549406 10.1126/science.abl5197 PMC7612735

[36] C. A. Xu , R. Lopez , E. Mehlman , J. Regier , M. I. Jordan , N. Yosef , Mol. Syst. Biol. 2019, 17, e9620. 10.15252/msb.20209620 PMC7829634 33491336

[37] Y. Cheng , X. Fan , J. Zhang , Y. Li , Commun. Biol. 2023, 6, 545. 37210444 10.1038/s42003-023-04928-6 PMC10199434

[38] T. Kipf , M. Welling , ArXiv 2016, *abs/1609.02907*.

[39] X. Fan , J. Liu , Y. Yang , C. Gu , Y. Han , B. Wu , Y. Jiang , G. Chen , P.‐A. Heng , Commun. Biol. 2024, 7, 1463. 39511415 10.1038/s42003-024-07154-w PMC11543810

[40] Z. Zhang , Y. Liu , M. Xiao , K. Wang , Y. Huang , J. Bian , R. Yang , F. Li , Brief. Bioinform. 2024, 25, bbae558. 39487083 10.1093/bib/bbae558 PMC11530284

[41] S.‐W. Tian , J.‐C. Ni , Y.‐T. Wang , C.‐H. Zheng , C.‐M. Ji , IEEE J Biomed Health Inform 2023.

[42] L. Tian , X. Dong , S. Freytag , K.‐A. L. Cao , S. Su , A. JalalAbadi , D. Amann‐Zalcenstein , T. S. Weber , A. Seidi , J. S. Jabbari , S. H. Naik , M. E. Ritchie , Nat. Methods 2019, 16, 479. 31133762 10.1038/s41592-019-0425-8

[43] Y. Xin , J. Kim , H. Okamoto , M. Ni , Y. Wei , C. Adler , A. J. Murphy , G. D. Yancopoulos , C. Lin , J. Gromada , Cell Metab. 2016, 24, 608. 27667665 10.1016/j.cmet.2016.08.018

[44] D. Usoskin , A. Furlan , S. Islam , H. Abdo , P. Lönnerberg , D. Lou , J. Hjerling‐Leffler , J. Haeggström , O. Kharchenko , P. V. Kharchenko , S. Linnarsson , P. Ernfors , Nat. Neurosci. 2015, 18, 145. 25420068 10.1038/nn.3881

[45] M. Baron , A. Veres , S. L. Wolock , A. L. Faust , R. Gaujoux , A. Vetere , J. H. Ryu , B. K. Wagner , S. S. Shen‐Orr , A. M. Klein , D. A. Melton , I. Yanai , Cell Syst. 2016, 3, 346. 27667365 10.1016/j.cels.2016.08.011 PMC5228327

[46] Å. Segerstolpe , A. Palasantza , P. Eliasson , E.‐M. Andersson , A.‐C. Andréasson , X. Sun , S. Picelli , A. Sabirsh , M. Clausen , M. K. Bjursell , D. M. Smith , M. Kasper , C. Ammala , R. Sandberg , Cell Metab. 2016, 24, 593. 27667667 10.1016/j.cmet.2016.08.020 PMC5069352

[47] M. J. Muraro , G. Dharmadhikari , D. Grün , N. Groen , T. Dielen , E. Jansen , L. Van Gurp , M. A. Engelse , F. Carlotti , E. J. De Koning , A. V. Oudenaarden , Cell Syst. 2016, 3, 385. 27693023 10.1016/j.cels.2016.09.002 PMC5092539

[48] N. Schaum , J. Karkanias , N. F. Neff , A. P. May , S. R. Quake , T. Wyss‐Coray , S. Darmanis , J. Batson , O. B. Botvinnik , M. B. Chen , S. Chen , F. Green , R. C. Jones , A. Maynard , L. Penland , A. O. Pisco , R. V. Sit , G. M. Stanley , J. T. Webber , F. Zanini , A. S. Baghel , I. Bakerman , I. Bansal , D. Berdnik , B. Bilen , D. G. Brownfield , C. Cain , M. Cho , G. Cirolia , S. D. Conley , et al., Nature 2018, 562, 367.30283141

[49] M. D. Young , T. J. Mitchell , F. A. Vieira Braga , M. G. Tran , B. J. Stewart , J. R. Ferdinand , G. Collord , R. A. Botting , D.‐M. Popescu , K. W. Loudon , R. V. Tormo , E. Stephenson , A. Cagan , S. J. Farndon , M. D. C. V. Herrera , C. Guzzo , N. Richoz , L. Mamanova , T. Aho , J. N. Armitage , A. C. P. Riddick , I. Mushtaq , S. Farrell , D. Rampling , J. Nicholson , A. Filby , J. Burge , S. Lisgo , P. H. Maxwell , et al., Science 2018, 361, 594. 30093597 10.1126/science.aat1699 PMC6104812

[50] K. Bach , S. Pensa , M. Grzelak , J. Hadfield , D. J. Adams , J. C. Marioni , W. T. Khaled , Nat. Commun. 2017, 8, 1. 29225342 10.1038/s41467-017-02001-5 PMC5723634

[51] M. Adam , A. S. Potter , S. S. Potter , Development 2017, 144, 3625. 28851704 10.1242/dev.151142 PMC5665481

[52] J. Jiang , C. Wang , R. Qi , H. Fu , Q. Ma , Iscience 2020, 23, 11. 10.1016/j.isci.2020.101769 PMC7674513 33241205

[53] X. Ren , W. Wen , X. Fan , W. Hou , B. Su , P. Cai , J. Li , Y. Liu , F. Tang , F. Zhang , X. Yang , J. He , W. Ma , J. He , P. Wang , Q. Cao , F. Chen , Y. Chen , X. Cheng , G. Deng , X. Deng , W. Ding , Y. Feng , R. Gan , C. Guo , W. Guo , S. He , C. Jiang , J. Liang , Y. M. Li , et al., Cell 2021, 184, 1895. 33657410 10.1016/j.cell.2021.01.053 PMC7857060

[54] J. Peng , B.‐F. Sun , C.‐Y. Chen , J.‐Y. Zhou , Y.‐S. Chen , H. Chen , L. Liu , D. Huang , J. Jiang , G. S. Cui , Y. Yang , W. Wang , D. Guo , M. Dai , J. Guo , T. Zhang , Q. Liao , Y. Liu , Y. L. Zhao , D. L. Han , Y. Zhao , Y. G. Yang , W. Wu , Cell Res. 2019, 29, 725. 31273297 10.1038/s41422-019-0195-y PMC6796938

[55] L. Tosti , Y. Hang , O. Debnath , S. Tiesmeyer , T. Trefzer , K. Steiger , F. W. Ten , S. Lukassen , S. Ballke , A. A. Kühl , S. Spieckermann , R. Bottino , N. Ishaque , W. Weichert , S. K. Kim , R. Eils , C. Conrad , Gastroenterology 2021, 160, 1330. 33212097 10.1053/j.gastro.2020.11.010

[56] J. B. Kang , A. Nathan , K. Weinand , F. Zhang , N. Millard , L. Rumker , D. B. Moody , I. Korsunsky , S. Raychaudhuri , Nat. Commun. 2021, 12, 5890. 34620862 10.1038/s41467-021-25957-x PMC8497570

[57] Z. Ma , N. K. Lytle , B. Chen , N. Jyotsana , S. W. Novak , C. J. Cho , L. Caplan , O. Ben‐Levy , A. C. Neininger , D. T. Burnette , V. Q. Trinh , M. C. B. Tan , E. A. Patterson , R. A. E. Drigo , R. R. Giraddi , C. Ramos , A. L. Means , I. Matsumoto , U. Manor , J. C. Mills , J. R. Goldenring , K. S. Lau , G. M. Wahl , Gastroenterology 2022, 162, 604. 34695382 10.1053/j.gastro.2021.10.027 PMC8792222

[58] F. Yang , W. Wang , F. Wang , Y. Fang , D. Tang , J. Huang , H. Lu , J. Yao , Nat. Mach. Intell. 2022, 4, 852.

[59] F. A. Wolf , P. Angerer , F. J. Theis , Genome Biol. 2018, 19, 1. 29409532 10.1186/s13059-017-1382-0 PMC5802054

[60] T. Stuart , A. Butler , P. Hoffman , C. Hafemeister , E. Papalexi , W. M. Mauck , Y. Hao , M. Stoeckius , P. Smibert , R. Satija , cell 2019, 177, 1888. 31178118 10.1016/j.cell.2019.05.031 PMC6687398

[61] M. D. Luecken , M. Büttner , K. Chaichoompu , A. Danese , M. Interlandi , M. F. Müller , D. C. Strobl , L. Zappia , M. Dugas , M. Colomé‐Tatché , F. J. Theis , Nat. Methods 2022, 19, 41. 34949812 10.1038/s41592-021-01336-8 PMC8748196

[62] K. Xu , C. Li , Y. Tian , T. Sonobe , K. ichi Kawarabayashi , S. Jegelka , ArXiv 2018, *abs/1806.03536*.

[63] J. Zhu , Y. Yan , L. Zhao , M. Heimann , L. Akoglu , D. Koutra , arXiv: Learning 2020.

[64] R. Jiang , T. Sun , D. Song , J. J. Li , Genome Biol. 2022, 23, 31. 35063006 10.1186/s13059-022-02601-5 PMC8783472

[65] D. Duvenaud , J. Wang , J. Jacobsen , K. Swersky , M. Norouzi , W. Grathwohl , International Conference on Learning Representations (ICLR) 2020.

[66] Q. Wu , Y. Chen , C. Yang , J. Yan , ICLR 2023.

[67] Y. Song , D. Wang , Proceedings of the 28th ACM SIGKDD Conference on Knowledge Discovery and Data Mining , ACM, New York, USA 2022, pp. 1635–1645.

