# Supplementary material for: scHeteroNet: A Heterophily‐Aware Graph Neural Network for Accurate Cell Type Annotation and Novel Cell Detection
Source: Adv Sci (Weinh). 2025 Mar 5;12(16):2412095. doi: 10.1002/advs.202412095 (PMC12021051; doi:10.1002/advs.202412095)
Supplement: Supplementary file 1 — Supporting Information [file ADVS-12-2412095-s001.pdf]

ADVANCED SCIENCE
Open Access

Supporting Information

for *Adv. Sci.*, DOI 10.1002/advs.202412095

scHeteroNet: A Heterophily-Aware Graph Neural Network for Accurate Cell Type Annotation and Novel Cell Detection

Jiacheng Liu, Xingyu Fan*, Chunbin Gu*, Yaodong Yang, Bian Wu, Guangyong Chen*, Chang-Yu Hsieh* and Pheng-Ann Heng*

Supplementary Information for: *scHeteroNet*: A Heterophily-Aware Graph Neural Network for Accurate Cell Type Annotation and Novel Cell Detection

Jiacheng Liu[1*†], Xingyu Fan[1†], Chunbin Gu[1*], Yaodong Yang[1], Bian Wu[2], Guangyong Chen[2*], Chang-Yu Hsieh[3*], Pheng-Ann Heng[1]

[1]Department of Computer Science and Engineering, The Chinese University of Hong Kong, Hong Kong, China.
[2]Zhejiang Lab, Hangzhou, China.
[3]College of Pharmaceutical Sciences, Zhejiang University, Hangzhou, China.

*Corresponding author(s). E-mail(s): jcliu@cse.cuhk.edu.hk; cbgu@cuhk.edu.hk; gychen@zhejianglab.com; kimhsieh@zju.edu.cn;
Contributing authors: xyfan@link.cuhk.edu.hk; yangyaodong@link.cuhk.edu.hk; wub@zhijianglab.com; pheng@cse.cuhk.edu.hk;
†These authors contributed equally to this work.

Appendix A Dataset details

We rigorously assessed *scHeteroNet*'s efficacy across a comprehensive array of scRNA-seq datasets that were sequenced using diverse protocols and originated from various organs. We collected most of the datasets from a benchmark pipeline [1], where their datasets are stored in the Zenodo repository (https://zenodo.org/records/3357167). Specifically, we collected six datasets from this pipeline, including Xin [2], Baron Mouse [3], Segerstolpe [4], Baron Human [3], Muraro [5], and TM [6]. Additionally, we collected 10x_5cl and CelSeq2_5cl from [7], which are derived from up to five distinct cancer cell lines. We also gathered 10x_LMuscle, 10x_Bladder, Sq_LMuscle,

Table A1: Overview of the datasets for evaluation in *scHeteroNet*.

Dataset	Organ	Cell	Gene	Class	Platform	Reference
10x_5cl	*Homo* lung	3,803	11,778	5	10*x Genomics*	[7]
Xin	*Homo* pancreas	1,449	33,889	4	SMARTer	[2]
Darmanis	*Mus* neuron	466	22,088	9	SMARTer	[13]
Baron Mouse	*Mus* pancreas	1,886	14,861	13	inDrop	[3]
Segerstolpe	*Homo* pancreas	2,133	22,757	13	Smart-seq2	[4]
Baron Human	*Homo* pancreas	8,569	17,499	14	inDrop	[3]
Muraro	*Homo* pancreas	2,122	19,046	9	CEL-seq2	[5]
TM	*Mus musculus*	54,865	19,791	55	10*x Genomics*	[6]
CelSeq2_5cl	*Homo* lung	570	12,627	5	CEL-Seq2	[7]
10x_LMuscle	*Mus* Limb Muscle	3,909	23,341	6	10*x Genomics*	[8]
10x_Bladder	*Mus* Bladder	2,500	23,341	4	10*x Genomics*	[8]
Sq_LMuscle	*Mus* Limb Muscle	1,090	23,341	6	Smart-seq2	[8]
Sq_Diaphragm	*Mus* Diaphragm	870	23,341	5	Smart-seq2	[8]
Sq_Heart	*Mus* Heart	4,365	23,341	8	Smart-seq2	[8]
Young	*Homo* Kidney	5,685	33,658	11	10*x Genomics*	[11]
Bach	*Homo* Gammary Gland	23,184	19,965	8	10*x Genomics*	[12]
Adam	*Mus* Kidney	3,660	23,797	8	Drop-seq	[14]
AD_ECortex	*Homo* Entorhinal Cortex	42,528	19,965	8	10*x Genomics*	[9]
Covid_Saliva	*Homo* Saliva	14,502	27,714	13	10*x Genomics*	[10]
Covid_SP	*Homo* Plasma, Saliva	14,990	27,714	8	10*x Genomics*	[10]

Sq_Diaphragm, and Sq_Heart from [8] to explore *scHeteroNet*'s robustness across various sequencing techniques and organs. Furthermore, AD ECortex [9], Covid Saliva, and Covid Saliva_Plasma [10] were collected to evaluate *scHeteroNet*'s performance on disease-specific and complex datasets. We also collected Young [11], Batch [12], Darmanis [13], and Adam [14] to further assess *scHeteroNet*'s performance across additional datasets. Initially, these datasets were utilized for annotating cell types in heterophily cell graphs. Subsequently, *scHeteroNet* employed the same datasets to validate its competence in identifying novel cells. Detailed information regarding each scRNA-seq dataset, including their respective organs, cell numbers, gene numbers, cell type numbers, and protocols, is provided in Table A1.

Appendix B Results on annotating cell types and identifying novel cells

For annotating cell types and identifying novel cells, we mainly used four evaluation metrics (including Accuracy (Table B2), F1-Score (Table B3), FPR (Table B5), and AUROC (Table B4)). All of these results were evaluated for five times independently. And we have documented all results and put them into the following table. For annotating cell types, we assessed each method's performance using accuracy and F1-score as metrics. The results revealed that *scHeteroNet* consistently surpassed the other methods, achieving higher mean accuracy and mean F1-scores across the datasets.

For identifying novel cells, specifically the Area Under the Receiver Operating Characteristic (AUROC) and the False Positive Rate (FPR) were used as metrics. And we also evaluated performance of *scHeteroNet* on those datasets using the Precision and Recall as metrics (Shown in Table B6 and B7).

Appendix C Impact of the topology of cell graphs

In scHeteroNet, we construct the cell graph using the KNN method, where each cell is connected to its K nearest neighbors based on gene expression similarity. The value of K is a critical parameter that determines the connectivity and structure of the resulting graph. A small value of K may result in a sparse graph with missing relationships, while a large value of K may lead to an overly dense graph with many potentially irrelevant connections.

The optimal choice of K depends on the specific characteristics of the dataset, such as the number of cells, the complexity of the cellular heterogeneity, and the level of noise in the gene expression data. A suitable K value should strike a balance between capturing the essential relationships among cells and avoiding the inclusion of spurious connections.

To investigate the impact of K on scHeteroNet's performance, we conducted experiments with varying K values (1, 3, 5, 7, 9, and 20) on 20 different datasets. The experimental results, as shown in Fig. C1 and C2, reveal several interesting patterns. Firstly, across the majority of datasets, scHeteroNet's performance is generally robust to the number of K, and for some datasets, it improves as K increases from 1 to 5. This suggests that incorporating information from a moderate number of nearest neighbors is beneficial for capturing the essential relationships among cells and improving the model's predictive power. Secondly, the performance tends to plateau or slightly decline when K is further increased beyond 5 or 7 (e.g., in Darmanis). This indicates that including too many neighbors may introduce noise or irrelevant connections, potentially hindering the model's ability to learn meaningful representations. Thirdly, the optimal K value varies depending on the specific dataset and the evaluation metric considered. For example, in the 10x 5cl dataset, the best performance in terms of FPR, AUROC, and Accuracy is achieved when K is around 7, while for the Darmanis dataset, the peak performance is observed at $K = 5$ for most metrics. Finally, some datasets, such as Xin and Muraro, exhibit a less pronounced impact of K on the model's performance. This suggests that the influence of graph topology may be less critical in certain biological contexts or when dealing with specific data characteristics.

These experimental results highlight the importance of carefully selecting the K value when constructing the cell graph for scHeteroNet. While our default setting of K=5 provides a good starting point, it is recommended to tune this parameter based on the specific characteristics of the dataset and the biological question at hand. The optimal K value should strike a balance between capturing the essential relationships among cells and avoiding the inclusion of noisy or irrelevant connections.

In conclusion, the experimental results demonstrate that the topology of the cell graph, as determined by the choice of K in the KNN method, has a different impact on

Table B2: Comparison of methods on accuracy. There are 220 experiments where each one was evaluating for five times. And values represent mean accuracy and standard deviation over the five results.

Dataset	scHeteroNet	ACTINN	CellTypeist	scmap-cell	scmap-cluster	scANVI	scBalance	GCN	scGraphFormer	scSimGCL	scGCC
10x_5cl	**1.0 ± 0.0**	**1.0 ± 0.0**	**1.0 ± 0.0**	**1.0 ± 0.0**	**1.0 ± 0.0**	**1.0 ± 0.0**	**1.0 ± 0.0**	**1.0 ± 0.0**	**1.0 ± 0.0**	**1.0 ± 0.0**	0.3616 ± 0.0228
Xin	0.9986 ± 0.0032	0.9749 ± 0.0268	0.9693 ± 0.0088	0.9929 ± 0.0067	**1.0 ± 0.0**	0.9943 ± 0.0041	0.9979 ± 0.0032	0.9986 ± 0.0032	0.9957 ± 0.003	0.9993 ± 0.0016	0.6012 ± 0.0236
Darmanis	**0.9643 ± 0.0317**	0.8528 ± 0.0565	0.8818 ± 0.044	0.8826 ± 0.023	0.893 ± 0.0441	0.9001 ± 0.0224	0.8866 ± 0.03	0.9114 ± 0.0447	0.9576 ± 0.0371	0.8751 ± 0.0433	0.2794 ± 0.0166
Baron Mouse	**0.9929 ± 0.0083**	0.9717 ± 0.0123	0.9554 ± 0.0094	0.9744 ± 0.0081	0.9837 ± 0.0058	0.9788 ± 0.0113	0.9902 ± 0.0053	0.975 ± 0.0138	0.9886 ± 0.0059	0.9875 ± 0.006	0.7084 ± 0.0198
Segerstolpe	**0.9924 ± 0.0054**	0.9356 ± 0.0825	0.9751 ± 0.0077	0.9637 ± 0.0057	0.9895 ± 0.0052	0.989 ± 0.0055	0.9857 ± 0.0046	0.9781 ± 0.0125	0.989 ± 0.0043	0.9866 ± 0.0032	0.5888 ± 0.0087
Baron Human	**0.9917 ± 0.0046**	0.9592 ± 0.016	0.9708 ± 0.0027	0.9793 ± 0.004	0.9717 ± 0.0027	0.9712 ± 0.0103	0.9861 ± 0.002	0.9854 ± 0.0061	0.9736 ± 0.0036	0.9854 ± 0.0032	0.3334 ± 0.0041
Muraro	**0.989 ± 0.0086**	0.9733 ± 0.0064	0.97 ± 0.008	0.9532 ± 0.0088	0.97 ± 0.009	0.9762 ± 0.0067	0.9771 ± 0.0083	0.9685 ± 0.0129	0.9662 ± 0.0135	0.979 ± 0.0054	0.5041 ± 0.0215
TM	0.968 ± 0.0021	0.8479 ± 0.0252	0.9635 ± 0.0012	0.9648 ± 0.0013	0.8892 ± 0.0021	0.7851 ± 0.0225	0.9698 ± 0.0023	0.9662 ± 0.0023	0.9043 ± nan	**0.9785 ± 0.0015**	0.7404 ± 0.0047
CelSeq2_5cl	**1.0 ± 0.0**	**1.0 ± 0.0**	**1.0 ± 0.0**	**1.0 ± 0.0**	**1.0 ± 0.0**	**1.0 ± 0.0**	**1.0 ± 0.0**	**1.0 ± 0.0**	**1.0 ± 0.0**	**1.0 ± 0.0**	0.3315 ± 0.07
10x_LMuscle	0.9981 ± 0.0016	0.9986 ± 0.0014	0.9986 ± 0.001	0.9981 ± 0.0007	0.9895 ± 0.0059	0.9992 ± 0.0012	**0.9995 ± 0.0008**	0.9939 ± 0.0012	0.9983 ± 0.0023	0.9992 ± 0.0008	0.6482 ± 0.0304
Sq_LMuscle	**0.9972 ± 0.0042**	0.9829 ± 0.0087	0.9782 ± 0.0073	0.9877 ± 0.0073	0.9914 ± 0.004	0.9905 ± 0.0059	0.9877 ± 0.0072	0.9933 ± 0.008	0.9914 ± 0.004	0.9725 ± 0.0162	0.501 ± 0.0369
Sq_Diaphragm	0.9976 ± 0.0033	0.994 ± 0.0042	0.9904 ± 0.0056	0.9964 ± 0.0033	0.9976 ± 0.0033	0.994 ± 0.0042	0.9964 ± 0.0033	0.9976 ± 0.0033	**0.9988 ± 0.0028**	0.9976 ± 0.0033	0.5369 ± 0.0307
Sq_Heart	0.9915 ± 0.0045	0.9753 ± 0.0295	0.9811 ± 0.0083	0.9903 ± 0.0021	0.9875 ± 0.0025	0.9845 ± 0.0067	**0.9922 ± 0.0022**	0.9901 ± 0.0036	0.9848 ± 0.0045	0.9917 ± 0.0022	0.9804 ± 0.0041
Young	**0.9845 ± 0.0068**	0.8951 ± 0.0494	0.9521 ± 0.0062	0.9274 ± 0.0132	0.8364 ± 0.0146	0.9614 ± 0.0043	0.9699 ± 0.0071	0.9735 ± 0.01	0.9429 ± 0.0171	0.9596 ± 0.0057	0.4441 ± 0.0143
Bach	**0.9933 ± 0.0025**	0.965 ± 0.008	0.9767 ± 0.002	0.9854 ± 0.001	0.9823 ± 0.0019	0.9906 ± 0.0017	0.988 ± 0.0056	0.9928 ± 0.0018	0.9657 ± 0.0094	0.9908 ± 0.0004	0.7849 ± 0.0034
Adam	**0.9842 ± 0.0083**	0.9693 ± 0.0089	0.9722 ± 0.0024	0.8343 ± 0.0034	0.9029 ± 0.0116	0.9713 ± 0.0084	0.9725 ± 0.0099	0.9707 ± 0.0135	0.9517 ± 0.0289	0.9734 ± 0.0074	0.1813 ± 0.0226
10x_Bladder	0.9992 ± 0.0011	0.9979 ± 0.0014	0.9967 ± 0.0011	**1.0 ± 0.0**	0.9979 ± 0.0021	0.9967 ± 0.0031	0.9984 ± 0.0017	**1.0 ± 0.0**	0.9934 ± 0.0017	0.9988 ± 0.0011	0.9701 ± 0.0047
AD_ECortex	**0.9807 ± 0.0018**	0.978 ± 0.0024	0.9695 ± 0.0023	0.9618 ± 0.003	0.9723 ± 0.0021	0.9785 ± 0.0018	0.9765 ± 0.0029	0.9795 ± 0.0017	0.9761 ± 0.0021	0.9771 ± 0.0024	0.7563 ± 0.0053
Covid_Saliva	**0.7941 ± 0.0166**	0.7437 ± 0.0093	0.6973 ± 0.0078	0.6743 ± 0.0144	0.561 ± 0.008	0.7401 ± 0.0073	0.6762 ± 0.0242	0.7705 ± 0.0303	0.7471 ± 0.0072	0.7514 ± 0.0086	0.6611 ± 0.0078
Covid_SP	**0.8056 ± 0.0186**	0.7519 ± 0.0115	0.711 ± 0.0099	0.6853 ± 0.0186	0.5864 ± 0.0112	0.749 ± 0.0174	0.689 ± 0.0266	0.7919 ± 0.0327	0.7653 ± 0.008	0.7637 ± 0.0044	0.6631 ± 0.0069

Table B3: Comparison of methods on F1-score. There are 220 experiments where each one was evaluating for five times. And values represent mean accuracy and standard deviation over the five results.

Dataset	scHeteroNet	ACTINN	CellTypeist	scmap-cell	scmap-cluster	scANVI	scBalance	GCN	scGraphFormer	scSimGCL	scGCC
10x_5cl	**1.0 ± 0.0**	**1.0 ± 0.0**	**1.0 ± 0.0**	**1.0 ± 0.0**	**1.0 ± 0.0**	**1.0 ± 0.0**	**1.0 ± 0.0**	**1.0 ± 0.0**	**1.0 ± 0.0**	**1.0 ± 0.0**	0.1327 ± 0.0063
Xin	0.9949 ± 0.0115	0.8599 ± 0.1727	0.9082 ± 0.0167	0.98 ± 0.0172	**1.0 ± 0.0**	0.9869 ± 0.0089	0.9944 ± 0.0079	0.9949 ± 0.0115	0.9872 ± 0.0094	0.9984 ± 0.0036	0.2502 ± 0.0062
Darmanis	**0.9587 ± 0.0354**	0.7987 ± 0.0476	0.8278 ± 0.0345	0.8531 ± 0.0521	0.8682 ± 0.0576	0.8857 ± 0.0229	0.8516 ± 0.0208	0.9023 ± 0.0387	0.9517 ± 0.0391	0.8677 ± 0.024	0.0546 ± 0.0025
Baron Mouse	**0.9877 ± 0.0147**	0.8953 ± 0.064	0.8733 ± 0.0212	0.9484 ± 0.0144	0.9679 ± 0.017	0.9393 ± 0.0563	0.9814 ± 0.0123	0.972 ± 0.0241	0.9804 ± 0.0155	0.9733 ± 0.0203	0.2987 ± 0.0176
Segerstolpe	**0.9711 ± 0.0285**	0.8293 ± 0.1069	0.8713 ± 0.0091	0.9172 ± 0.0232	0.9576 ± 0.0275	0.9587 ± 0.0265	0.9409 ± 0.0203	0.9383 ± 0.036	0.9523 ± 0.0365	0.9461 ± 0.0172	0.1884 ± 0.0018
Baron Human	**0.98 ± 0.0125**	0.7196 ± 0.0651	0.9066 ± 0.0294	0.9448 ± 0.0123	0.9652 ± 0.0057	0.7621 ± 0.0501	0.978 ± 0.0052	0.9655 ± 0.008	0.8207 ± 0.0342	0.9706 ± 0.0218	0.09 ± 0.0024
Muraro	**0.9863 ± 0.0125**	0.9636 ± 0.013	0.9628 ± 0.0083	0.9363 ± 0.0151	0.9617 ± 0.01	0.9715 ± 0.0107	0.9717 ± 0.0115	0.9602 ± 0.0154	0.9528 ± 0.0246	0.9733 ± 0.0092	0.1827 ± 0.0066
TM	0.8485 ± 0.0096	0.3712 ± 0.0564	0.8745 ± 0.0088	0.9086 ± 0.0035	0.8172 ± 0.0057	0.2581 ± 0.0249	0.9251 ± 0.0055	0.8587 ± 0.0073	0.5453 ± nan	**0.9451 ± 0.0056**	0.3346 ± 0.0015
CelSeq2_5cl	**1.0 ± 0.0**	**1.0 ± 0.0**	**1.0 ± 0.0**	**1.0 ± 0.0**	**1.0 ± 0.0**	**1.0 ± 0.0**	**1.0 ± 0.0**	**1.0 ± 0.0**	**1.0 ± 0.0**	**1.0 ± 0.0**	0.1236 ± 0.0207
10x_LMuscle	0.9977 ± 0.0018	0.9976 ± 0.0024	0.9984 ± 0.0012	0.9977 ± 0.001	0.9871 ± 0.0068	0.999 ± 0.0015	**0.9994 ± 0.0009**	0.9922 ± 0.0013	0.9979 ± 0.0029	0.9991 ± 0.0009	0.3131 ± 0.0096
Sq_LMuscle	**0.9974 ± 0.0039**	0.9703 ± 0.0205	0.9675 ± 0.0066	0.9797 ± 0.0088	0.984 ± 0.0072	0.9861 ± 0.0053	0.9811 ± 0.0091	0.9895 ± 0.0128	0.9836 ± 0.0071	0.9564 ± 0.0223	0.1334 ± 0.0064
Sq_Diaphragm	0.9954 ± 0.0063	0.9906 ± 0.0054	0.9851 ± 0.0091	0.9961 ± 0.0047	0.9969 ± 0.005	0.9901 ± 0.0059	0.9946 ± 0.0058	0.9969 ± 0.005	**0.9977 ± 0.0051**	0.9969 ± 0.005	0.1746 ± 0.0066
Sq_Heart	0.9657 ± 0.0093	0.8853 ± 0.1457	0.9387 ± 0.0336	0.9651 ± 0.0093	0.9569 ± 0.0105	0.8597 ± 0.0583	0.9643 ± 0.0084	**0.97 ± 0.0078**	0.9241 ± 0.0174	0.9682 ± 0.0162	0.826 ± 0.0069
Young	**0.9839 ± 0.0075**	0.7604 ± 0.1534	0.943 ± 0.0142	0.9168 ± 0.0216	0.8545 ± 0.0197	0.9383 ± 0.0377	0.9672 ± 0.0093	0.971 ± 0.0119	0.9234 ± 0.0231	0.958 ± 0.0069	0.1406 ± 0.0066
Bach	**0.9816 ± 0.0064**	0.8385 ± 0.0554	0.939 ± 0.0077	0.9584 ± 0.0045	0.9496 ± 0.0055	0.973 ± 0.0026	0.9646 ± 0.0132	0.9796 ± 0.0045	0.8369 ± 0.0472	0.9707 ± 0.001	0.5547 ± 0.0077
Adam	**0.9836 ± 0.0085**	0.9679 ± 0.0091	0.971 ± 0.0018	0.8217 ± 0.005	0.9097 ± 0.0096	0.9699 ± 0.0084	0.9716 ± 0.0101	0.97 ± 0.0136	0.9534 ± 0.0244	0.973 ± 0.0074	0.0511 ± 0.0144
10x_Bladder	0.9955 ± 0.0062	0.9871 ± 0.0085	0.9915 ± 0.0066	**1.0 ± 0.0**	0.9939 ± 0.0077	0.9875 ± 0.0084	0.9967 ± 0.0058	**1.0 ± 0.0**	0.9562 ± 0.0111	0.9992 ± 0.0008	0.6565 ± 0.0017
AD_ECortex	**0.9826 ± 0.0019**	0.98 ± 0.0025	0.9726 ± 0.0022	0.9643 ± 0.0031	0.9748 ± 0.002	0.981 ± 0.0019	0.9793 ± 0.0029	0.9812 ± 0.0017	0.9781 ± 0.0021	0.9797 ± 0.0025	0.7532 ± 0.0054
Covid_Saliva	0.459 ± 0.0262	0.2048 ± 0.0077	0.4298 ± 0.0185	0.4542 ± 0.033	0.4605 ± 0.0172	0.263 ± 0.0399	0.4905 ± 0.0212	**0.5077 ± 0.0598**	0.2845 ± 0.0251	0.4826 ± 0.035	0.1646 ± 0.0038
Covid_SP	0.5097 ± 0.0432	0.2615 ± 0.0105	0.4737 ± 0.0175	0.472 ± 0.0402	0.5086 ± 0.04	0.3601 ± 0.052	0.5435 ± 0.0275	**0.5628 ± 0.046**	0.3279 ± 0.012	0.5111 ± 0.0252	0.2067 ± 0.0028

Table B4: Comparison of methods on AUROC. There are 220 experiments where each one was evaluating for five times. And values represent mean accuracy and standard deviation over the five results.

Dataset	scHeteroNet	ACTINN	CellTypeist	scmap-cell	scmap-cluster	scANVI	scBalance	GCN	scGraphFormer	scSimGCL	scGCC
10x_5cl	0.9995 ± 0.0008	0.8575 ± 0.1697	0.5299 ± 0.0163	0.5519 ± 0.0078	0.5012 ± 0.0	0.9998 ± 0.0001	0.9999 ± 0.0001	0.9141 ± 0.0168	**1.0 ± 0.0**	0.9928 ± 0.0013	0.8707 ± 0.0028
Xin	0.9929 ± 0.0056	0.9539 ± 0.0653	0.7988 ± 0.034	0.7273 ± 0.018	0.5174 ± 0.0146	0.9908 ± 0.0044	**0.9976 ± 0.0008**	0.9881 ± 0.0028	**0.9976 ± 0.0015**	0.994 ± 0.0013	0.6679 ± 0.0
Darmanis	0.9174 ± 0.0357	0.9281 ± 0.0469	0.4987 ± 0.0009	0.7716 ± 0.0919	0.4982 ± 0.001	0.921 ± 0.0288	0.7167 ± 0.0649	0.3667 ± 0.0957	0.9141 ± 0.0233	**0.9393 ± 0.0173**	0.678 ± 0.0163
Baron Mouse	0.9847 ± 0.0133	0.8319 ± 0.2502	0.5019 ± 0.0202	0.877 ± 0.063	0.4998 ± 0.0002	0.9751 ± 0.0097	0.9785 ± 0.0081	0.9524 ± 0.0239	**0.9871 ± 0.0086**	0.9829 ± 0.0049	0.7218 ± 0.018
Segerstolpe	0.9866 ± 0.0102	0.9735 ± 0.0377	0.5802 ± 0.0901	0.5661 ± 0.1532	0.4993 ± 0.0002	0.9772 ± 0.0135	0.9895 ± 0.0102	0.9006 ± 0.0806	**0.9935 ± 0.0078**	0.992 ± 0.0039	0.9926 ± 0.0003
Baron Human	0.9977 ± 0.0021	0.9869 ± 0.0103	**0.9991 ± 0.0004**	0.7337 ± 0.0522	0.4994 ± 0.0001	0.9867 ± 0.0092	0.9941 ± 0.0013	0.9817 ± 0.0161	0.9947 ± 0.0005	0.9963 ± 0.001	0.422 ± 0.0005
Muraro	**0.989 ± 0.007**	0.9636 ± 0.019	0.5564 ± 0.0639	0.8269 ± 0.0564	0.5135 ± 0.0212	0.9749 ± 0.0095	0.9793 ± 0.0079	0.8794 ± 0.1088	0.9623 ± 0.0087	0.9841 ± 0.0107	0.9541 ± 0.0001
TM	0.9705 ± 0.0092	0.9341 ± 0.0199	0.9113 ± 0.0316	0.8033 ± 0.1824	0.4995 ± 0.0001	0.9662 ± 0.0184	0.8667 ± 0.0914	0.945 ± 0.0437	**0.982 ± nan**	0.9542 ± 0.0121	0.8873 ± 0.0039
CelSeq2_5cl	0.992 ± 0.0062	0.9004 ± 0.1189	0.5 ± 0.0	0.5231 ± 0.0154	0.5 ± 0.0	0.9996 ± 0.0008	0.9998 ± 0.0003	0.9131 ± 0.0078	**1.0 ± 0.0**	0.9619 ± 0.0069	0.4398 ± 0.0129
10x_LMuscle	0.993 ± 0.0013	0.9769 ± 0.0189	0.7951 ± 0.059	0.794 ± 0.1287	0.5142 ± 0.0019	0.9946 ± 0.0011	**0.9962 ± 0.0005**	0.9623 ± 0.0203	0.987 ± 0.0014	0.9855 ± 0.0046	0.7117 ± 0.0103
Sq_LMuscle	**0.9983 ± 0.0024**	0.9922 ± 0.0019	0.5051 ± 0.008	0.592 ± 0.0136	0.4997 ± 0.0003	0.9693 ± 0.0061	0.9897 ± 0.0024	0.838 ± 0.1637	0.9907 ± 0.0024	0.9862 ± 0.0042	0.5263 ± 0.0067
Sq_Diaphragm	0.959 ± 0.0694	0.9739 ± 0.0177	0.5053 ± 0.0087	0.6466 ± 0.0218	0.5063 ± 0.0087	0.9945 ± 0.0034	0.9923 ± 0.0017	0.8159 ± 0.1781	**0.999 ± 0.0005**	0.9901 ± 0.0034	0.6092 ± 0.0001
Sq_Heart	0.9834 ± 0.0132	0.9569 ± 0.0378	0.6953 ± 0.1683	0.5411 ± 0.0686	0.4999 ± 0.0001	0.9799 ± 0.0057	**0.9926 ± 0.0036**	0.8911 ± 0.0416	0.9693 ± 0.0037	0.9553 ± 0.0247	0.9716 ± 0.0108
Young	0.9949 ± 0.0019	0.9584 ± 0.0229	**0.9978 ± 0.0008**	0.8866 ± 0.0254	0.5238 ± 0.006	0.9656 ± 0.0238	0.968 ± 0.0044	0.9651 ± 0.0213	0.9793 ± 0.0126	0.794 ± 0.0668	0.805 ± 0.0079
Bach	0.9392 ± 0.0233	0.7008 ± 0.1302	0.5651 ± 0.0418	0.4842 ± 0.0018	0.5003 ± 0.001	0.521 ± 0.0727	0.5945 ± 0.1818	0.603 ± 0.0413	0.7139 ± 0.0519	**0.9423 ± 0.02**	0.4315 ± 0.006
Adam	0.9544 ± 0.0031	0.9244 ± 0.0287	0.9486 ± 0.0099	0.6397 ± 0.016	0.5014 ± 0.0045	0.9411 ± 0.009	0.9591 ± 0.0178	0.8546 ± 0.014	**0.9596 ± 0.0104**	0.8428 ± 0.0308	0.4579 ± 0.0662
10x_Bladder	0.9892 ± 0.006	0.9835 ± 0.0155	0.5452 ± 0.0472	0.6268 ± 0.0331	0.5 ± 0.0	0.9915 ± 0.0062	0.9915 ± 0.0076	0.8922 ± 0.0305	0.9931 ± 0.0014	**0.9936 ± 0.004**	0.9684 ± 0.0008
AD_ECortex	**0.9815 ± 0.0022**	0.929 ± 0.0747	0.9335 ± 0.0125	0.7547 ± 0.0148	0.5087 ± 0.0013	0.9305 ± 0.01	0.9386 ± 0.0039	0.9427 ± 0.0013	0.9767 ± 0.0013	0.9343 ± 0.009	0.7802 ± 0.0016
Covid_Saliva	**0.9918 ± 0.0022**	0.9874 ± 0.0028	0.8986 ± 0.0369	0.6598 ± 0.0587	0.498 ± 0.0005	0.8471 ± 0.0808	0.5792 ± 0.0523	0.9542 ± 0.0171	0.9578 ± 0.0036	0.7674 ± 0.0139	0.8891 ± 0.0047
Covid_SP	**0.9917 ± 0.0025**	0.9886 ± 0.0024	0.9259 ± 0.0383	0.7211 ± 0.0297	0.5128 ± 0.0214	0.9164 ± 0.0649	0.5828 ± 0.0469	0.9685 ± 0.0059	0.9838 ± 0.0029	0.8189 ± 0.0408	0.844 ± 0.0061

Table B5: Comparison of methods on FPR. There are 220 experiments where each one was evaluating for five times. And values represent mean accuracy and standard deviation over the five results.

Dataset	scHeteroNet	ACTINN	CellTypeist	scmap-cell	scmap-cluster	scANVI	scBalance	GCN	scGraphFormer	scSimGCL	scGCC
10x_5cl	0.0014 ± 0.0031	0.4421 ± 0.429	0.9402 ± 0.0326	0.8963 ± 0.0156	0.9977 ± 0.0	**0.0 ± 0.0**	**0.0 ± 0.0**	0.1308 ± 0.0227	**0.0 ± 0.0**	0.0262 ± 0.0108	0.7206 ± 0.0264
Xin	0.0087 ± 0.0194	0.2565 ± 0.3691	0.4 ± 0.0587	0.5261 ± 0.0357	0.9652 ± 0.0292	0.0391 ± 0.0389	**0.0 ± 0.0**	0.0522 ± 0.0476	0.0217 ± 0.0	0.0348 ± 0.0248	1.0 ± 0.0
Darmanis	0.6125 ± 0.2911	0.475 ± 0.3295	1.0 ± 0.0	0.8375 ± 0.26	1.0 ± 0.0	0.65 ± 0.1369	0.925 ± 0.028	0.95 ± 0.028	0.5375 ± 0.1801	**0.4375 ± 0.2165**	1.0 ± 0.0
Baron Mouse	0.0714 ± 0.1597	0.4714 ± 0.5041	0.9714 ± 0.0391	0.2 ± 0.1174	1.0 ± 0.0	0.1 ± 0.0814	0.1 ± 0.0814	0.4286 ± 0.2766	**0.0429 ± 0.0639**	0.1 ± 0.0639	1.0 ± 0.0
Segerstolpe	0.0933 ± 0.13	0.1733 ± 0.2385	0.8267 ± 0.1801	1.0 ± 0.0	1.0 ± 0.0	0.12 ± 0.1966	0.0133 ± 0.0298	0.5333 ± 0.3232	0.0133 ± 0.0298	**0.0 ± 0.0**	**0.0 ± 0.0**
Baron Human	**0.0 ± 0.0**	0.0615 ± 0.0843	**0.0 ± 0.0**	0.5077 ± 0.1032	1.0 ± 0.0	0.0615 ± 0.0644	**0.0 ± 0.0**	0.1077 ± 0.15	**0.0 ± 0.0**	**0.0 ± 0.0**	0.9231 ± 0.0
Muraro	**0.0381 ± 0.0213**	0.1619 ± 0.1704	0.8857 ± 0.1287	0.6952 ± 0.4187	0.9714 ± 0.0426	0.1333 ± 0.0976	**0.0381 ± 0.0213**	0.4381 ± 0.3038	0.1905 ± 0.1684	0.0952 ± 0.0825	0.2286 ± 0.0213
TM	0.1167 ± 0.0745	0.7167 ± 0.3685	0.1583 ± 0.0543	0.3333 ± 0.3644	1.0 ± 0.0	0.175 ± 0.2271	0.4583 ± 0.2534	0.3083 ± 0.1431	**0.0833 ± nan**	0.3333 ± 0.1443	0.5083 ± 0.0349
CelSeq2_5cl	0.0246 ± 0.0354	0.4185 ± 0.3975	1.0 ± 0.0	0.9538 ± 0.0308	1.0 ± 0.0	**0.0 ± 0.0**	**0.0 ± 0.0**	0.1415 ± 0.0334	**0.0 ± 0.0**	0.2646 ± 0.0398	1.0 ± 0.0
10x_LMuscle	0.0357 ± 0.0092	0.124 ± 0.1198	0.4182 ± 0.1163	0.4058 ± 0.2568	0.9708 ± 0.004	**0.0136 ± 0.0081**	0.0143 ± 0.0054	0.1162 ± 0.0666	0.0429 ± 0.0036	0.0513 ± 0.0148	0.9786 ± 0.0233
Sq_LMuscle	**0.0 ± 0.0**	**0.0 ± 0.0**	0.9943 ± 0.0128	0.8 ± 0.0286	1.0 ± 0.0	0.2057 ± 0.0843	**0.0 ± 0.0**	0.5371 ± 0.4926	**0.0 ± 0.0**	0.0286 ± 0.0286	1.0 ± 0.0
Sq_Diaphragm	0.1742 ± 0.3174	0.2 ± 0.1873	0.9871 ± 0.0177	0.6968 ± 0.0433	0.9871 ± 0.0177	**0.0 ± 0.0**	0.0129 ± 0.0177	0.5613 ± 0.3625	**0.0 ± 0.0**	0.0258 ± 0.0144	1.0 ± 0.0
Sq_Heart	0.05 ± 0.0931	0.3214 ± 0.4195	0.5929 ± 0.3403	0.9 ± 0.1369	1.0 ± 0.0	0.0786 ± 0.0687	**0.0 ± 0.0**	0.3929 ± 0.1406	0.0286 ± 0.0466	0.3143 ± 0.2089	0.1071 ± 0.2396
Young	**0.0 ± 0.0**	0.2767 ± 0.1953	**0.0 ± 0.0**	0.73 ± 0.0506	0.95 ± 0.0118	0.17 ± 0.1977	0.2133 ± 0.0639	0.2067 ± 0.1494	0.1333 ± 0.1054	0.74 ± 0.1367	0.8333 ± 0.0656
Bach	0.5034 ± 0.1856	0.9125 ± 0.091	0.8332 ± 0.0729	0.9826 ± 0.0034	0.9985 ± 0.0021	0.9253 ± 0.0819	0.9019 ± 0.1583	0.9525 ± 0.0243	0.926 ± 0.0391	**0.3351 ± 0.1104**	1.0 ± 0.0
Adam	0.2098 ± 0.0228	0.4366 ± 0.2143	**0.0884 ± 0.027**	0.7714 ± 0.0631	0.9929 ± 0.0093	0.2982 ± 0.0506	0.1875 ± 0.0797	0.6607 ± 0.0501	0.1955 ± 0.0392	0.5429 ± 0.0713	0.9982 ± 0.0024
10x_Bladder	0.0351 ± 0.0691	0.0386 ± 0.056	0.9263 ± 0.0923	0.7439 ± 0.0663	1.0 ± 0.0	0.0175 ± 0.0215	0.0211 ± 0.0314	0.2982 ± 0.0541	0.0246 ± 0.0235	0.0175 ± 0.0392	**0.0 ± 0.0**
AD_ECortex	**0.0586 ± 0.0023**	0.2836 ± 0.2971	0.1471 ± 0.0259	0.4452 ± 0.0294	0.9822 ± 0.0026	0.1118 ± 0.018	0.2362 ± 0.0103	0.1655 ± 0.0038	0.0918 ± 0.005	0.317 ± 0.0566	0.8427 ± 0.0043
Covid_Saliva	**0.0 ± 0.0**	0.019 ± 0.0261	0.2381 ± 0.101	0.8095 ± 0.126	1.0 ± 0.0	0.7619 ± 0.1347	0.8381 ± 0.1096	0.1619 ± 0.0543	0.3333 ± 0.0337	0.7429 ± 0.0543	0.6571 ± 0.0398
Covid_SP	0.0095 ± 0.0213	**0.0 ± 0.0**	0.2095 ± 0.0426	0.7714 ± 0.0398	0.9714 ± 0.0426	0.4762 ± 0.3611	0.8 ± 0.0706	0.1429 ± 0.0583	0.019 ± 0.0261	0.581 ± 0.0621	0.7524 ± 0.0213

Table B6: Comparison of methods on Precision. There are 220 experiments where each one was evaluating for five times. And values represent mean accuracy and standard deviation over the five results.

Dataset	scHeteroNet	ACTINN	CellTypeist	scmap-cell	scmap-cluster	scANVI	scBalance	GCN	scGraphFormer	scSimGCL	scGCC
10x_5cl	**1.0 ± 0.0**	**1.0 ± 0.0**	**1.0 ± 0.0**	**1.0 ± 0.0**	**1.0 ± 0.0**	**1.0 ± 0.0**	**1.0 ± 0.0**	**1.0 ± 0.0**	**1.0 ± 0.0**	**1.0 ± 0.0**	0.0904 ± 0.0057
Xin	0.9992 ± 0.0017	0.9203 ± 0.1581	0.9715 ± 0.0149	0.9962 ± 0.0035	**1.0 ± 0.0**	0.9914 ± 0.0084	0.9988 ± 0.0018	0.9992 ± 0.0017	0.9977 ± 0.0016	0.9996 ± 0.0009	0.2004 ± 0.0079
Darmanis	**0.965 ± 0.0328**	0.8916 ± 0.0257	0.8613 ± 0.0578	0.8763 ± 0.0563	0.8935 ± 0.0456	0.8967 ± 0.0199	0.882 ± 0.0427	0.9018 ± 0.0521	0.9598 ± 0.0364	0.9027 ± 0.023	0.0349 ± 0.0021
Baron Mouse	0.9908 ± 0.0112	0.8997 ± 0.0774	0.941 ± 0.0504	0.9744 ± 0.0128	0.9671 ± 0.0107	0.9426 ± 0.0671	0.9848 ± 0.013	0.9868 ± 0.007	**0.9938 ± 0.0053**	0.9792 ± 0.0193	0.2782 ± 0.043
Segerstolpe	0.9731 ± 0.0316	0.86 ± 0.1108	0.9226 ± 0.065	0.9156 ± 0.0182	0.9678 ± 0.0347	0.9738 ± 0.0361	**0.9841 ± 0.0015**	0.9356 ± 0.0318	0.963 ± 0.0402	0.9651 ± 0.0289	0.1515 ± 0.0024
Baron Human	**0.9929 ± 0.0058**	0.7351 ± 0.0478	0.945 ± 0.054	0.9767 ± 0.0103	0.9701 ± 0.0066	0.7494 ± 0.0563	0.9818 ± 0.0077	0.9863 ± 0.0084	0.8468 ± 0.05	0.9816 ± 0.0088	0.1059 ± 0.0045
Muraro	**0.9858 ± 0.0103**	0.963 ± 0.0096	0.9655 ± 0.0116	0.9593 ± 0.0089	0.9535 ± 0.0116	0.9693 ± 0.0113	0.9685 ± 0.0114	0.9638 ± 0.0171	0.9612 ± 0.0205	0.9715 ± 0.0111	0.1363 ± 0.0072
TM	0.914 ± 0.014	0.3913 ± 0.0453	0.9301 ± 0.0117	0.933 ± 0.0086	0.8113 ± 0.0088	0.2363 ± 0.0259	0.9185 ± 0.0085	0.9084 ± 0.011	0.5735 ± nan	**0.9578 ± 0.0076**	0.3599 ± 0.0126
CelSeq2_5cl	**1.0 ± 0.0**	**1.0 ± 0.0**	**1.0 ± 0.0**	**1.0 ± 0.0**	**1.0 ± 0.0**	**1.0 ± 0.0**	**1.0 ± 0.0**	**1.0 ± 0.0**	**1.0 ± 0.0**	**1.0 ± 0.0**	0.0829 ± 0.0175
10x_LMuscle	0.998 ± 0.0016	0.9974 ± 0.0026	0.998 ± 0.0012	0.9968 ± 0.0012	0.9819 ± 0.0088	**0.9995 ± 0.0007**	0.9994 ± 0.0009	0.9925 ± 0.0024	0.9991 ± 0.0013	0.999 ± 0.0011	0.2763 ± 0.0116
Sq_LMuscle	**0.999 ± 0.0015**	0.9699 ± 0.0173	0.9559 ± 0.0091	0.9739 ± 0.0131	0.9804 ± 0.0117	0.9878 ± 0.0083	0.9784 ± 0.012	0.987 ± 0.017	0.9795 ± 0.0118	0.9603 ± 0.0212	0.1002 ± 0.0074
Sq_Diaphragm	0.9926 ± 0.0102	0.991 ± 0.0089	0.9767 ± 0.0148	0.9946 ± 0.008	0.9956 ± 0.0084	0.9874 ± 0.0083	0.992 ± 0.0097	0.9956 ± 0.0084	**0.9962 ± 0.0086**	0.9956 ± 0.0084	0.1342 ± 0.0077
Sq_Heart	0.9654 ± 0.0175	0.891 ± 0.1621	0.9709 ± 0.0197	0.9578 ± 0.0071	0.9473 ± 0.013	0.8534 ± 0.0668	0.9611 ± 0.0076	0.9688 ± 0.0124	**0.9753 ± 0.0102**	0.9677 ± 0.0166	0.81 ± 0.0117
Young	**0.9863 ± 0.0092**	0.7886 ± 0.1818	0.9646 ± 0.0096	0.9227 ± 0.0173	0.8345 ± 0.0215	0.9359 ± 0.0389	0.9634 ± 0.0131	0.9737 ± 0.0112	0.9635 ± 0.0075	0.9588 ± 0.0046	0.1489 ± 0.0093
Bach	**0.9879 ± 0.0043**	0.8996 ± 0.0736	0.963 ± 0.0066	0.9552 ± 0.0056	0.947 ± 0.0078	0.977 ± 0.0023	0.9708 ± 0.0098	0.9842 ± 0.0051	0.9677 ± 0.0053	0.9726 ± 0.0045	0.6651 ± 0.0275
Adam	**0.9836 ± 0.0088**	0.969 ± 0.0086	0.9722 ± 0.0021	0.8923 ± 0.0056	0.9253 ± 0.0091	0.9706 ± 0.0082	0.9729 ± 0.0092	0.9721 ± 0.0126	0.9607 ± 0.0175	0.9736 ± 0.0069	0.0351 ± 0.0157
10x_Bladder	0.9994 ± 0.0008	0.9905 ± 0.0117	0.9858 ± 0.0121	**1.0 ± 0.0**	0.9986 ± 0.0014	0.9852 ± 0.0111	0.9989 ± 0.0012	**1.0 ± 0.0**	0.9955 ± 0.0012	0.9992 ± 0.0008	0.6467 ± 0.0032
AD_ECortex	**0.9835 ± 0.0017**	0.9809 ± 0.002	0.9774 ± 0.0015	0.97 ± 0.0025	0.9761 ± 0.0018	0.9804 ± 0.0014	0.9795 ± 0.0026	0.982 ± 0.0014	0.9781 ± 0.0019	0.9799 ± 0.0023	0.7879 ± 0.0042
Covid_Saliva	0.6171 ± 0.0944	0.2327 ± 0.0337	0.5461 ± 0.0419	0.5269 ± 0.0647	0.4952 ± 0.0237	0.2595 ± 0.043	0.5135 ± 0.0225	**0.6365 ± 0.1033**	0.3294 ± 0.0356	0.5542 ± 0.0453	0.201 ± 0.0091
Covid_SP	0.6489 ± 0.0868	0.2859 ± 0.0392	0.5697 ± 0.0212	0.5188 ± 0.0517	0.5239 ± 0.0397	0.3551 ± 0.0537	0.5829 ± 0.0435	**0.6736 ± 0.0698**	0.3922 ± 0.0188	0.5734 ± 0.0319	0.2375 ± 0.0066

Table B7: Comparison of methods on Recall. There are 220 experiments where each one was evaluating for five times. And values represent mean accuracy and standard deviation over the five results.

Dataset	scHeteroNet	ACTINN	CellTypeist	scmap-cell	scmap-cluster	scANVI	scBalance	GCN	scGraphFormer	scSimGCL	scGCC
10x_5cl	**1.0 ± 0.0**	**1.0 ± 0.0**	**1.0 ± 0.0**	**1.0 ± 0.0**	**1.0 ± 0.0**	**1.0 ± 0.0**	**1.0 ± 0.0**	**1.0 ± 0.0**	**1.0 ± 0.0**	**1.0 ± 0.0**	0.25 ± 0.0
Xin	0.9911 ± 0.0199	0.8562 ± 0.1649	0.8707 ± 0.0255	0.9666 ± 0.0277	**1.0 ± 0.0**	0.983 ± 0.014	0.9903 ± 0.0135	0.9911 ± 0.0199	0.9779 ± 0.0161	0.9972 ± 0.0062	0.3333 ± 0.0
Darmanis	**0.9587 ± 0.0343**	0.7732 ± 0.0495	0.8805 ± 0.0175	0.8573 ± 0.0505	0.87 ± 0.055	0.8986 ± 0.0173	0.8637 ± 0.0204	0.9156 ± 0.0293	0.9478 ± 0.0418	0.8592 ± 0.0277	0.125 ± 0.0
Baron Mouse	**0.9858 ± 0.0174**	0.8959 ± 0.0537	0.8699 ± 0.022	0.9338 ± 0.0184	0.9706 ± 0.0222	0.9399 ± 0.0468	0.9799 ± 0.0146	0.9612 ± 0.0344	0.9699 ± 0.0223	0.969 ± 0.0229	0.3507 ± 0.0117
Segerstolpe	**0.9696 ± 0.0261**	0.8269 ± 0.1106	0.8762 ± 0.0026	0.9464 ± 0.0265	0.9516 ± 0.0225	0.9511 ± 0.0201	0.9287 ± 0.0203	0.9471 ± 0.0289	0.9449 ± 0.0354	0.9372 ± 0.0116	0.2497 ± 0.0007
Baron Human	0.9713 ± 0.0169	0.7208 ± 0.0643	0.89 ± 0.0206	0.9287 ± 0.0188	0.9625 ± 0.0094	0.7821 ± 0.0407	**0.9756 ± 0.009**	0.9526 ± 0.0093	0.8031 ± 0.0264	0.9673 ± 0.0273	0.1202 ± 0.0039
Muraro	**0.9872 ± 0.0146**	0.9662 ± 0.0162	0.9617 ± 0.008	0.9241 ± 0.0214	0.9719 ± 0.0102	0.9747 ± 0.0103	0.976 ± 0.0115	0.9586 ± 0.0175	0.9472 ± 0.0277	0.976 ± 0.008	0.2806 ± 0.0044
TM	0.8263 ± 0.0108	0.3851 ± 0.05	0.8464 ± 0.0092	0.9038 ± 0.0062	0.8918 ± 0.0061	0.3033 ± 0.0213	**0.9427 ± 0.0044**	0.8367 ± 0.0087	0.552 ± nan	0.9368 ± 0.0042	0.3536 ± 0.002
CelSeq2_5cl	**1.0 ± 0.0**	**1.0 ± 0.0**	**1.0 ± 0.0**	**1.0 ± 0.0**	**1.0 ± 0.0**	**1.0 ± 0.0**	**1.0 ± 0.0**	**1.0 ± 0.0**	**1.0 ± 0.0**	**1.0 ± 0.0**	0.25 ± 0.0
10x_LMuscle	0.9974 ± 0.0023	0.9978 ± 0.0023	0.9989 ± 0.0013	0.9986 ± 0.0011	0.9927 ± 0.0045	0.9986 ± 0.0022	**0.9993 ± 0.0011**	0.992 ± 0.0022	0.9967 ± 0.0044	0.9992 ± 0.001	0.376 ± 0.0073
Sq_LMuscle	**0.996 ± 0.0061**	0.9723 ± 0.0255	0.9819 ± 0.0083	0.9869 ± 0.0069	0.9889 ± 0.0039	0.9853 ± 0.0045	0.9852 ± 0.007	0.9929 ± 0.0084	0.9889 ± 0.0039	0.9558 ± 0.028	0.2 ± 0.0
Sq_Diaphragm	0.9985 ± 0.0022	0.9907 ± 0.0095	0.9946 ± 0.003	0.9978 ± 0.0022	0.9983 ± 0.0024	0.9933 ± 0.0094	0.9974 ± 0.0025	0.9983 ± 0.0024	**0.9994 ± 0.0013**	0.9983 ± 0.0024	0.25 ± 0.0
Sq_Heart	0.9694 ± 0.0104	0.892 ± 0.1185	0.9169 ± 0.0404	**0.975 ± 0.0131**	0.9734 ± 0.012	0.8687 ± 0.049	0.9698 ± 0.0105	0.9725 ± 0.0133	0.9025 ± 0.0148	0.9703 ± 0.0166	0.8463 ± 0.0029
Young	**0.9819 ± 0.0062**	0.7532 ± 0.1293	0.9262 ± 0.0184	0.9196 ± 0.0229	0.8933 ± 0.0145	0.9436 ± 0.0374	0.9723 ± 0.0068	0.9688 ± 0.0125	0.8972 ± 0.0299	0.9586 ± 0.0099	0.195 ± 0.0041
Bach	**0.9758 ± 0.0091**	0.8228 ± 0.047	0.9208 ± 0.0123	0.9622 ± 0.0069	0.9546 ± 0.0041	0.9695 ± 0.0041	0.9613 ± 0.0202	0.9752 ± 0.0056	0.8199 ± 0.042	0.9692 ± 0.0041	0.5469 ± 0.0061
Adam	**0.9838 ± 0.0082**	0.9677 ± 0.009	0.9705 ± 0.002	0.8253 ± 0.0081	0.9077 ± 0.01	0.9699 ± 0.0085	0.9711 ± 0.0105	0.9689 ± 0.0135	0.9518 ± 0.0245	0.9727 ± 0.0079	0.1483 ± 0.0081
10x_Bladder	0.9919 ± 0.0112	0.9843 ± 0.0138	0.9977 ± 0.0008	**1.0 ± 0.0**	0.9896 ± 0.0137	0.9902 ± 0.0121	0.9947 ± 0.0102	**1.0 ± 0.0**	0.9265 ± 0.0172	0.9992 ± 0.0008	0.6667 ± 0.0
AD_ECortex	**0.9818 ± 0.0021**	0.9792 ± 0.0029	0.9683 ± 0.0027	0.9597 ± 0.0034	0.9735 ± 0.0023	**0.9818 ± 0.0023**	0.9792 ± 0.0033	0.9804 ± 0.002	0.9782 ± 0.0024	0.9795 ± 0.0027	0.7322 ± 0.0057
Covid_Saliva	0.4263 ± 0.026	0.2235 ± 0.0045	0.3848 ± 0.0168	0.4567 ± 0.0325	**0.5457 ± 0.0137**	0.2873 ± 0.0493	0.4919 ± 0.022	0.478 ± 0.0461	0.285 ± 0.024	0.4559 ± 0.0284	0.1745 ± 0.0042
Covid_SP	0.4792 ± 0.0384	0.2829 ± 0.0032	0.4348 ± 0.0181	0.4747 ± 0.0432	**0.5856 ± 0.036**	0.391 ± 0.0538	0.5434 ± 0.0203	0.5322 ± 0.0439	0.3283 ± 0.0117	0.4869 ± 0.0239	0.2157 ± 0.0029

scHeteroNet's prediction performance on different datasets. While a moderate number of neighbors (e.g., K=5 or 7) generally yields good results, the optimal K value may vary depending on the specific dataset and evaluation metric. Careful tuning of this parameter is crucial for constructing an informative graph that captures the essential relationships among cells while avoiding the inclusion of noisy or irrelevant connections.

Appendix D Comparing with large models

Given the increasing prominence of large language models in single-cell analysis, we conducted some preliminary comparisons between scBert and scHeteroNet across four widely-used pancreatic datasets: Xin[2], Segerstolpe[4], Muraro[5], and Baron Human[3]. The performance was also evaluated using four key metrics: Accuracy, F1-Score, AUROC, and FPR, as illustrated in Fig. C3. The results demonstrate that scHeteroNet consistently outperforms scBert across all metrics and datasets. First, scHeteroNet achieves superior accuracy and F1-scores across all datasets, with particularly notable improvements in the Xin and Baron Human datasets. The accuracy improvements range from 5-10 percentage points across different datasets. The AUROC scores for scHeteroNet are substantially higher than scBert across all datasets, with the most significant difference observed in the Muraro and Segerstolpe datasets where scHeteroNet achieves near-perfect AUROC scores while scBert's performance is notably lower. Finally, scHeteroNet shows much lower FPR in all four datasets which indicates scHeteroNet's superior ability to minimize false positive predictions, making it more reliable for practical applications where false positives could lead to misleading biological interpretations. The consistent superior performance of scHeteroNet can be attributed to its heterogeneous graph architecture, which better captures the complex relationships between cells compared to scBert's language model-based approach.

Appendix E Time and Space Complexity Analysis

To evaluate scHeteroNet's efficiency against other methods, we analyzed computational efficiency and memory requirements across varying dataset sizes (scGCC was excluded from this analysis due to its significantly longer runtime). Results are shown in Fig. E4 and Fig. E5. The datasets in Table A1 represent different scales of single-cell transcriptomic data, providing a comprehensive benchmark for performance assessment.

As shown in Fig. E4, most methods exhibit relatively efficient runtime performance, with scHeteroNet demonstrating moderate computational demands. While methods like ACTINN, CellTypeist, and scmap variants achieve faster execution times, and scANVI and scSimGCL show higher computational costs, scHeteroNet maintains a balanced performance profile that scales reasonably with increasing cell numbers. In terms of memory consumption (Fig. E5), we observe that scHeteroNet maintains one of the lowest memory footprints across all dataset sizes. This is particularly notable when compared to methods like scmap-cell and scANVI, which show substantially higher memory usage as the number of cells increases. The memory-efficient design of

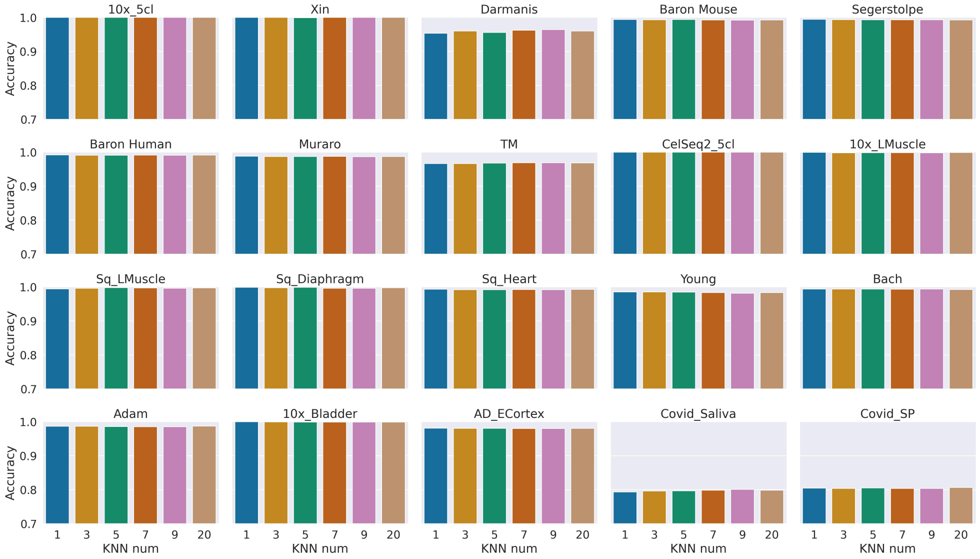

(a) Accuracy

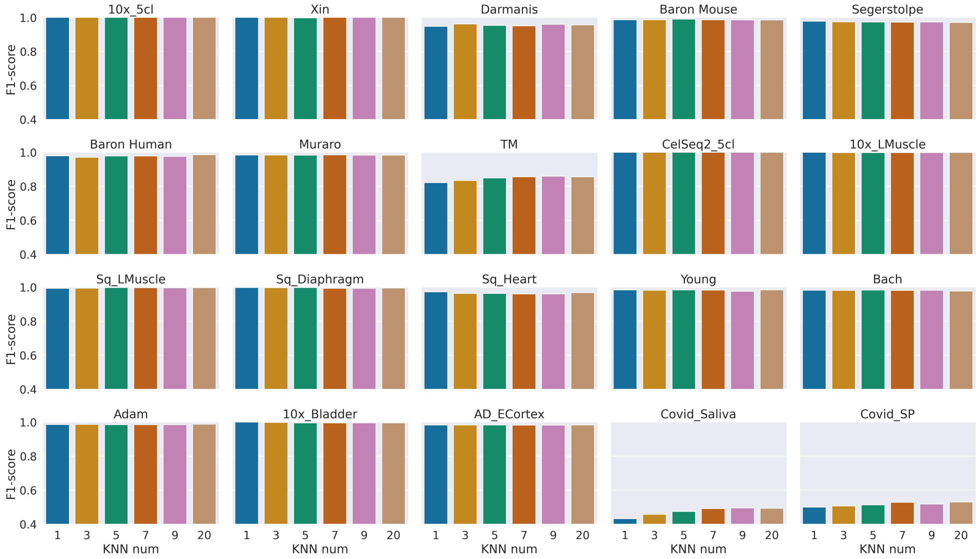

(b) F1 score

Fig. C1: Annotation performance with varying numbers of neighborhoods in KNN graph construction.

scHeteroNet makes it particularly suitable for analyzing large-scale single-cell datasets, where computational resources can be a limiting factor.

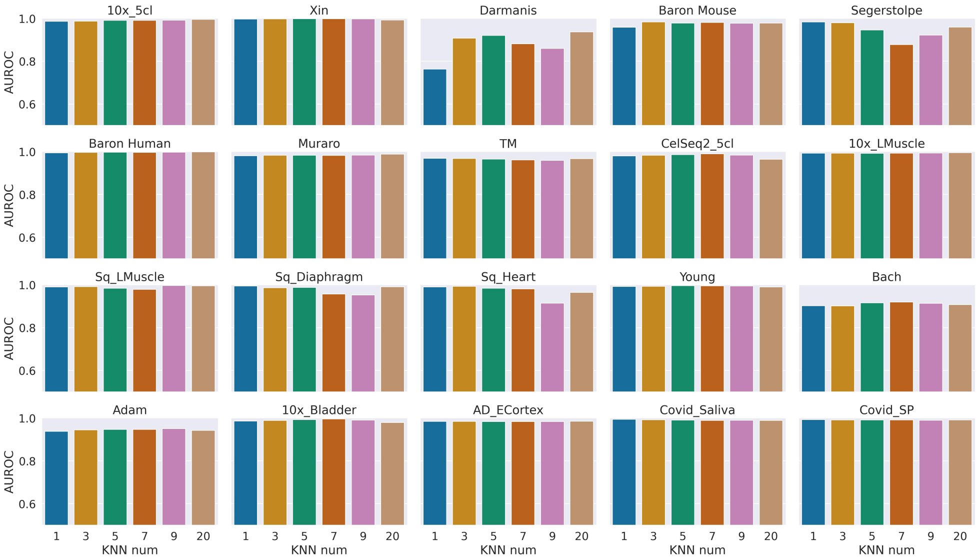

(a) AUROC

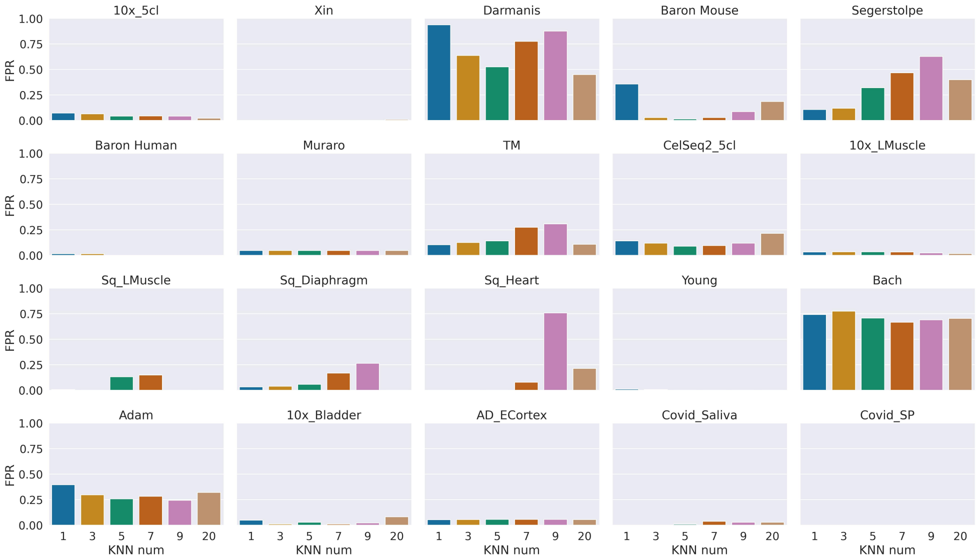

(b) FPR

Fig. C2: Novel cell detection performance with varying numbers of neighborhoods in KNN graph construction.

These benchmarks suggest that scHeteroNet achieves a favorable balance between computational efficiency and memory utilization, making it a practical choice for single-cell analysis tasks across various dataset scales.

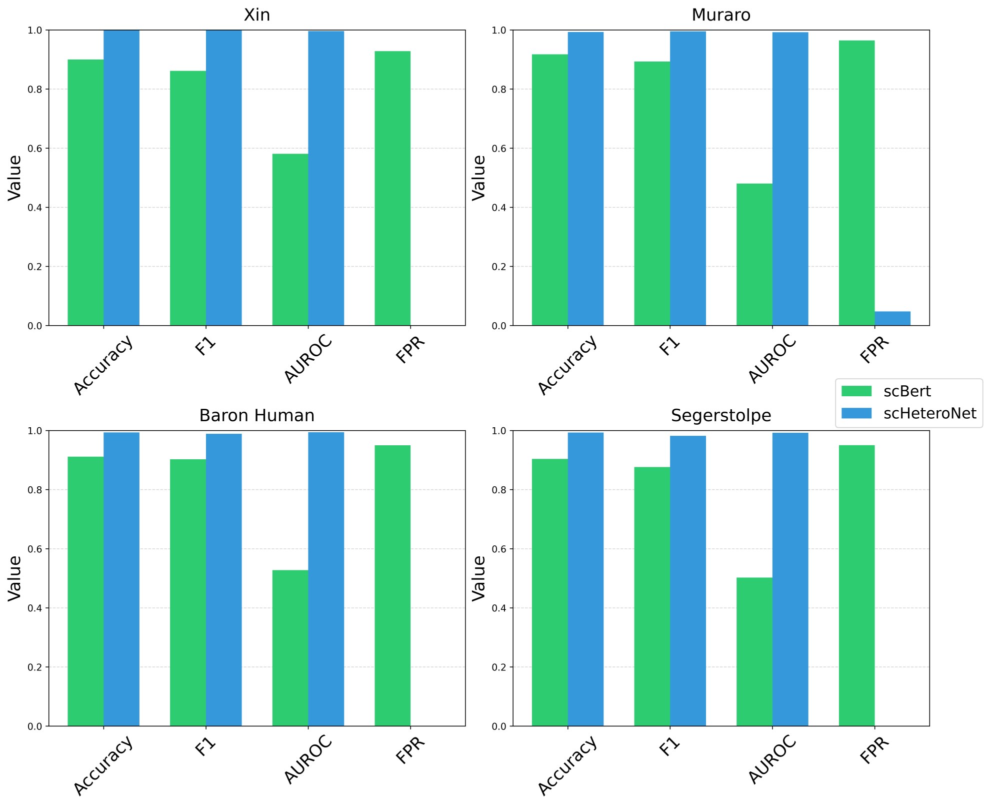

Fig. C3: Comparison between scBert and scHeteroNet using F1-Score, Accuracy, FPR and AUROC metrics across four widely-used pancreatic datasets (Xin, Muraro, Baron Human, and Segerstolpe).

Appendix F *scHeteroNet* enables accurate cell type annotation and novel cell detection in spatial transcriptomics

To showcase scHeteroNet's broader applicability beyond scRNA-seq, we tested its performance on spatial transcriptomics data using five samples (DLPFC_151676, DLPFC_151510, DLPFC_151670, DLPFC_151669, and DLPFC_151672) from the dorsolateral prefrontal cortex (DLPFC) dataset [15]. Spatial transcriptomics represents a distinct challenge as it preserves spatial information while capturing gene expression data, requiring methods that can effectively handle both aspects. Instead of the baselines for scRNA, we also include spaGCN [16] as a baseline which was specially designed for spatial transcriptomics.

Our comprehensive evaluation demonstrates scHeteroNet's superior performance across multiple metrics (**Figure E6**). In terms of accuracy, scHeteroNet achieves

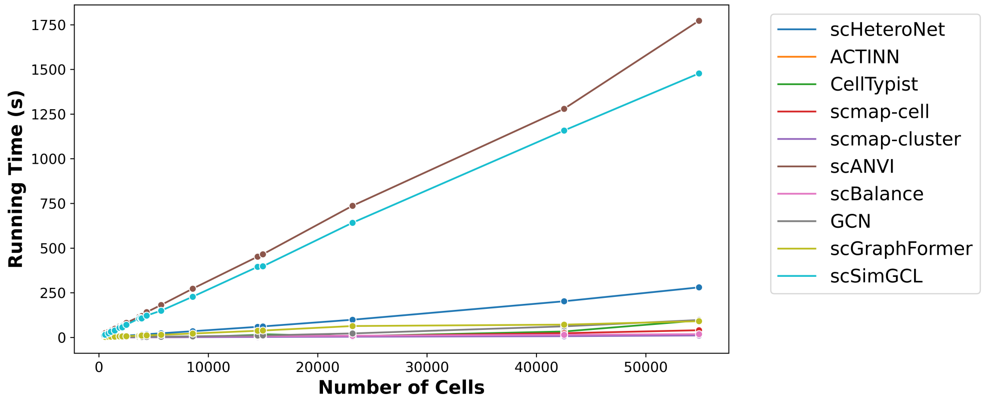

Fig. E4: Comparison of running time across different single-cell analysis methods with increasing cell numbers. The experiment evaluates the computational efficiency of ten state-of-the-art methods including scHeteroNet, ACTINN, CellTypeist, scmap-cell, scmap-cluster, scANVI, scBalance, GCN, scGraphFormer, and scSimGCL. The x-axis represents the number of cells ranging from 0 to 55,000 (from 20 datasets), while the y-axis shows the running time in seconds.

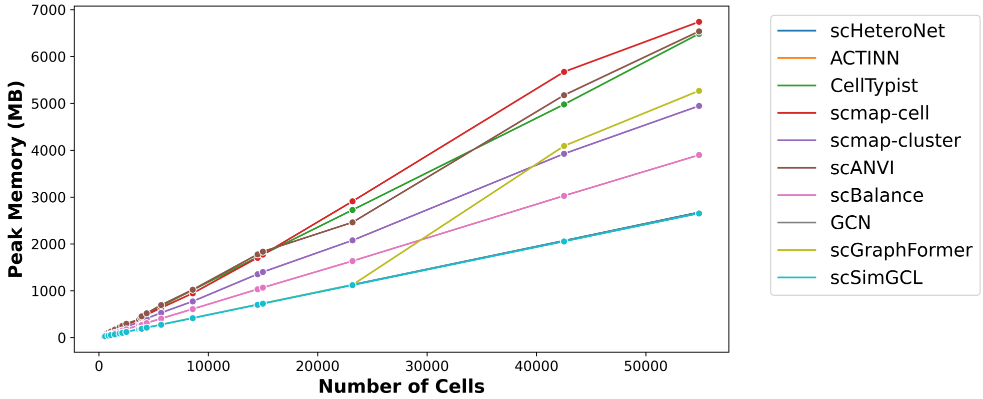

Fig. E5: Comparison of peak memory consumption across different single-cell analysis methods with varying cell numbers. The x-axis shows the number of cells (ranging from 0 to 55,000) from 20 datasets, while the y-axis displays the peak memory usage in megabytes (MB).

accuracy scores that are nearly 1.0 in different datasets (Figure 8a). This performance advantage is further supported by the F1-scores, where scHeteroNet maintained its leading position, indicating robust performance even with imbalanced cell type distributions (**Figure E6**b).

Particularly noteworthy is scHeteroNet's performance in novel cell detection within spatial contexts, as evidenced by the AUROC scores (**Figure E6**c) and

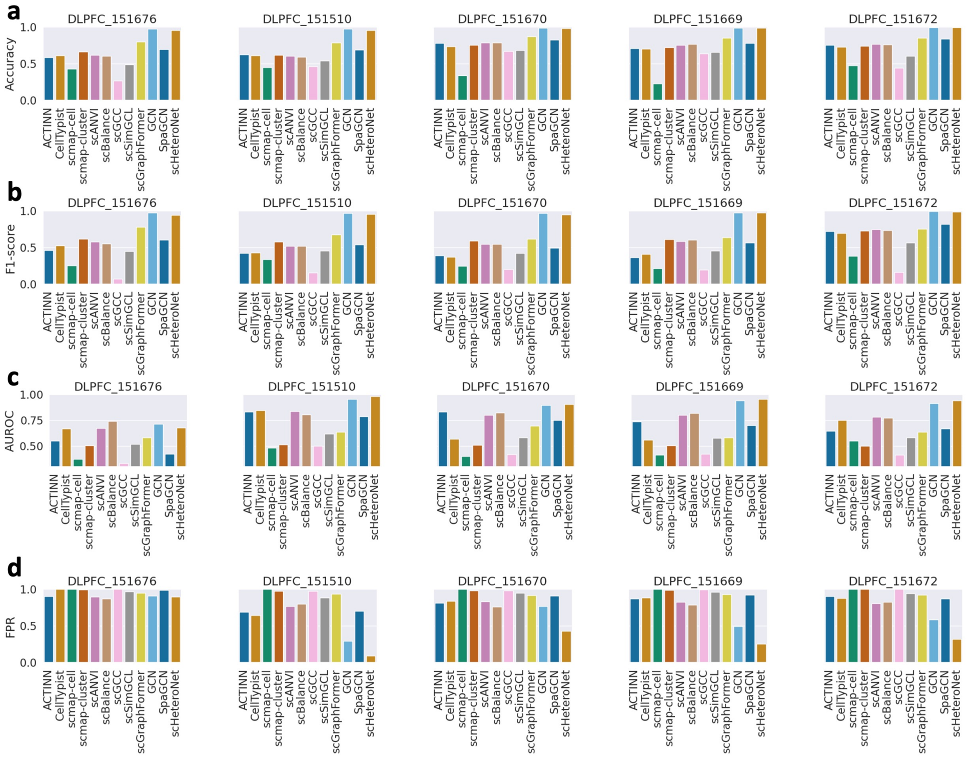

Fig. E6: *scHeteroNet* can accurately annotate cell types and detect novel cells in spatial transcriptomic. **a,** Accuracy comparisons between scHeteroNet and 11 single-cell annotation methods. **b,** F1-score comparisons between scHeteroNet and 11 single-cell annotation methods. **c,** AUROC comparisons between scHeteroNet and 11 single-cell annotation methods. **d,** FPR comparisons between scHeteroNet and 11 single-cell annotation methods.

the FPR score (**Figure** E6d). The model achieves the lowest FPR which further validates scHeteroNet's reliability. Specifically, in DLPFC_151510, scHeteroNet demonstrated remarkable FPR reduction, achieving rates below 0.1, while other methods typically showed FPR values above 0.6. This significant improvement in FPR indicates scHeteroNet's superior ability to minimize false positive identifications while maintaining high detection sensitivity.

The comparative analysis reveals that while some existing methods like scGraphFormer and SpaGCN perform reasonably well in certain metrics, they often show inconsistent performance across different samples. In contrast, scHeteroNet maintains stable and superior performance across all samples and metrics. Besides, we also found the graph construct is always a homogeneous graph which makes the graph a critical resource for cell annotation, this makes models like GCN outperform other methods.

These results demonstrate scHeteroNet's successful extension to spatial transcriptomics analysis, establishing it as a versatile tool capable of handling both traditional scRNA-seq and spatial transcriptomics data. The model's robust performance across different metrics and samples suggests its potential as a reliable method for analyzing complex spatial transcriptomics datasets, offering new possibilities for understanding tissue architecture and cellular organization.

Appendix G Integration with Contrastive Learning

We also conduct an experiment to incorporate contrastive learning into our single-cell gene expression analysis framework to learn robust cell representations that are invariant to stochastic gene expression fluctuations. This approach enhances the model's ability to capture essential biological features while being resistant to technical noise.

G.1 Gene Expression View Generation

Given the original gene expression matrix $\mathbf{X} \in \mathbb{R}^{n \times d}$, where n is the number of cells and d is the number of genes, we generate two distinct views through random masking. For each cell i, we create two random mask vectors:

$$\mathbf{m}_i^1, \mathbf{m}_i^2 \sim \text{Bernoulli}(p) \tag{G1}$$

where p represents the retention probability. The two views are obtained through element-wise multiplication:

$$\mathbf{X}^1 = \mathbf{X} \odot \mathbf{M}^1, \quad \mathbf{X}^2 = \mathbf{X} \odot \mathbf{M}^2 \tag{G2}$$

G.2 Representation Learning

Each view is processed through our encoder network to obtain latent representations:

$$\mathbf{z}^1 = f_\theta(\mathbf{X}^1, \mathbf{A}), \quad \mathbf{z}^2 = f_\theta(\mathbf{X}^2, \mathbf{A}) \tag{G3}$$

where f_θ represents the encoder network and $\mathbf{A}$ is the cell-cell adjacency matrix (can be 1-hop or multi-hops).

G.3 Contrastive Loss

We employ the InfoNCE loss [17] to maximize mutual information between different views of the same cell:

$$\mathcal{L}_{\text{contrast}} = -\mathbb{E}_i \left[\log \frac{\exp(\text{sim}(\mathbf{z}_i^1, \mathbf{z}_i^2)/\tau)}{\sum_{j=1}^{n} \exp(\text{sim}(\mathbf{z}_i^1, \mathbf{z}_j^2)/\tau)} \right] \tag{G4}$$

where $\text{sim}(\cdot, \cdot)$ denotes cosine similarity and τ is a temperature parameter.

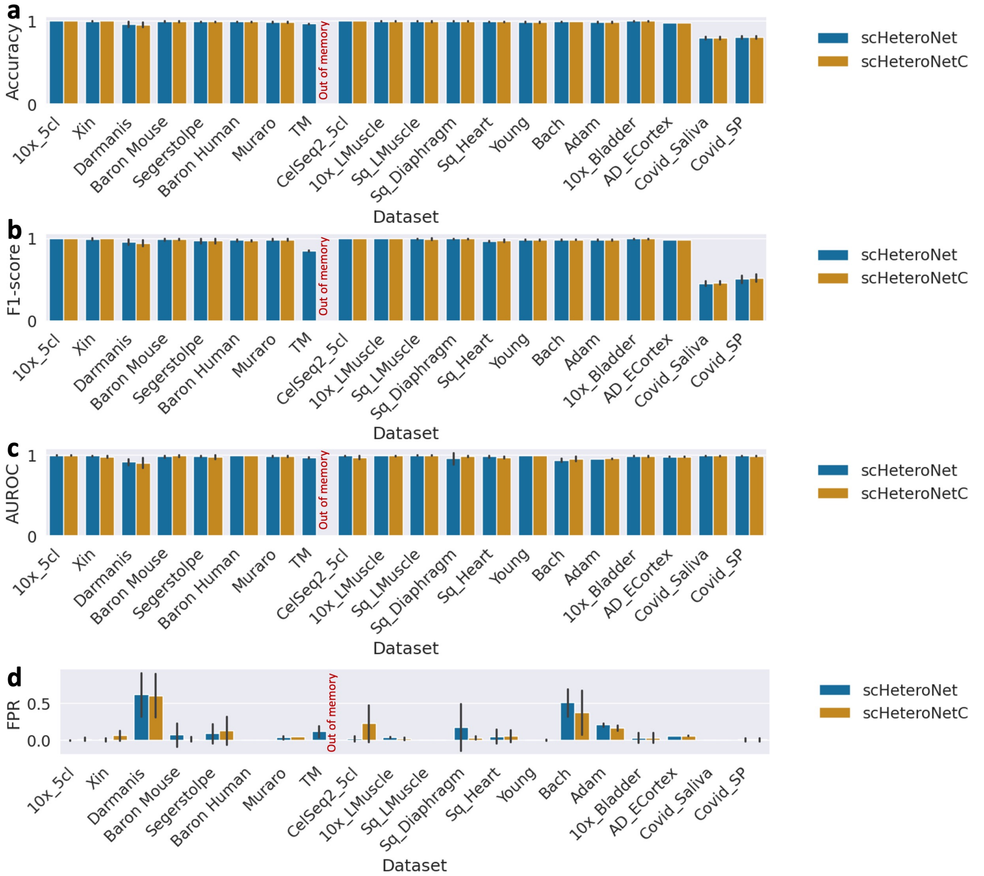

Fig. G7: Comparison of *scHeteroNet* and its contrastive learning-based variant (scHeteroNetC).

G.4 Joint Optimization

The final training objective combines supervised classification loss, ZINB reconstruction loss, and contrastive loss:

$$\mathcal{L}_{\text{total}} = \mathcal{L}_{\text{sup}} + \lambda_1 \mathcal{L}_{\text{zinb}} + \lambda_2 \mathcal{L}_{\text{contrast}} \tag{G5}$$

where λ_1 and λ_2 are weighting coefficients for the ZINB and contrastive losses, respectively. This multi-objective optimization enables the model to learn both discriminative and generalizable features from single-cell gene expression data.

G.5 Experiment Results

In Figure G7, a comprehensive comparison is made between scHeteroNet and scHeteroNetC, where scHeteroNetC is scHeteroNet with an additional contrastive

learning objective. The bar chart in Figure G7a-d shows that the accuracy, F1-score, AUROC and FPR of scHeteroNet and scHeteroNetC are very close across different datasets. There is no significant difference in these two algorithms, indicating that the addition of the contrastive learning objective does not enhance the performance of the model. Despite this, it is observed that scHeteroNetC requires more memory compared to scHeteroNet. In fact, when running on a 4090 GPU, scHeteroNetC encounters out-of-memory errors in TM dataset. This significant drawback makes scHeteroNetC less practical for implementation.

Based on the results presented in Figure G7, adding a contrastive learning objective to scHeteroNet does not yield clear advantages in terms of performance metrics such as accuracy, AUROC, F1-score, and FPR. Moreover, it leads to increased memory consumption. Therefore, it has been decided that the contrastive learning objective should not be included in the current implementation. However, future research could focus on devising better contrastive learning objectives to potentially overcome these limitations.

References

[1] Abdelaal, T. *et al.* A comparison of automatic cell identification methods for single-cell rna sequencing data. *Genome biology* **20**, 1–19 (2019).

[2] Xin, Y. *et al.* Rna sequencing of single human islet cells reveals type 2 diabetes genes. *Cell metabolism* **24**, 608–615 (2016).

[3] Baron, M. *et al.* A single-cell transcriptomic map of the human and mouse pancreas reveals inter-and intra-cell population structure. *Cell systems* **3**, 346–360 (2016).

[4] Segerstolpe, Å. *et al.* Single-cell transcriptome profiling of human pancreatic islets in health and type 2 diabetes. *Cell metabolism* **24**, 593–607 (2016).

[5] Muraro, M. J. *et al.* A single-cell transcriptome atlas of the human pancreas. *Cell systems* **3**, 385–394 (2016).

[6] Schaum, N. *et al.* Single-cell transcriptomics of 20 mouse organs creates a tabula muris: The tabula muris consortium. *Nature* **562**, 367 (2018).

[7] Tian, L. *et al.* Benchmarking single cell rna-sequencing analysis pipelines using mixture control experiments. *Nature Methods* **16**, 479 – 487 (2019).

[8] Schaum, N. *et al.* Single-cell transcriptomics of 20 mouse organs creates a tabula muris. *Nature* **562**, 367 – 372 (2018).

[9] Jiang, J., Wang, C., Qi, R., Fu, H. & Ma, Q. scread: a single-cell rna-seq database for alzheimer's disease. *Iscience* **23** (2020).

[10] Ren, X. *et al.* Covid-19 immune features revealed by a large-scale single-cell transcriptome atlas. *Cell* **184**, 1895–1913 (2021).

[11] Young, M. D. *et al.* Single-cell transcriptomes from human kidneys reveal the cellular identity of renal tumors. *science* **361**, 594–599 (2018).

[12] Bach, K. *et al.* Differentiation dynamics of mammary epithelial cells revealed by single-cell rna sequencing. *Nature communications* **8**, 1–11 (2017).

[13] Usoskin, D. *et al.* Unbiased classification of sensory neuron types by large-scale single-cell rna sequencing. *Nature neuroscience* **18**, 145–153 (2015).

[14] Adam, M., Potter, A. S. & Potter, S. S. Psychrophilic proteases dramatically reduce single-cell rna-seq artifacts: a molecular atlas of kidney development. *Development* **144**, 3625–3632 (2017).

[15] Maynard, K. R. *et al.* Transcriptome-scale spatial gene expression in the human dorsolateral prefrontal cortex. *Nature neuroscience* **24**, 425–436 (2021).

[16] Hu, J. *et al.* Spagcn: Integrating gene expression, spatial location and histology to identify spatial domains and spatially variable genes by graph convolutional network. *Nature methods* **18**, 1342–1351 (2021).

[17] Oord, A. v. d., Li, Y. & Vinyals, O. Representation learning with contrastive predictive coding. *arXiv preprint arXiv:1807.03748* (2018).
